# Weighing the evidence on costs and benefits of polygenic risk-based approaches in clinical practice: A systematic review of economic evaluations

**DOI:** 10.1016/j.ajhg.2025.05.012

**Published:** 2025-06-12

**Authors:** Leonardo Maria Siena, Valentina Baccolini, Marianna Riccio, Annalisa Rosso, Giuseppe Migliara, Antonio Sciurti, Claudia Isonne, Jessica Iera, Francesco Pierri, Carolina Marzuillo, Corrado De Vito, Giuseppe La Torre, Paolo Villari

**Affiliations:** 1 Department of Public Health and Infectious Diseases, Sapienza University of Rome, Rome, Italy; 2 Department of Translational and Precision Medicine, Sapienza University of Rome, Rome, Italy; 3 Department of Life Sciences, Health, and Health Professions, Link Campus University, Rome, Italy

**Keywords:** polygenic risk score, PRS, polygenic risk, economic evaluation, systematic review

## Abstract

Polygenic risk scores (PRSs) represent a promising innovation in the context of precision health, but their benefits for patients and healthcare systems remain unclear. This systematic review examined the methods used to quantify the costs and benefits of PRS-based approaches across different healthcare contexts, summarizing current evidence and identifying challenges. A systematic search of three databases was conducted, and full economic evaluations related to any intervention based on polygenic risk stratification strategies were included (PROSPERO CRD42023442780). Quality was assessed using the Quality of Health Economic Studies instrument. Studies were grouped into three categories (cancer, cardiovascular disease, and other diseases), and key methodological features and characteristics were extracted. A total of 24 cost-utility analyses of generally high quality were included: 16 studies focused on cancer, five on cardiovascular disease, and three on other diseases. Studies on cancer mainly aimed to optimize screening programs, while in the other fields, PRSs were mostly used to refine eligibility for preventive therapies. Analyses were robust, but they mostly relied on hypothetical cohorts, had limited generalizability, paid insufficient attention to implementation aspects—including the delivery model—and considered only clinical benefits. Despite a positive trend toward cost effectiveness following PRS implementation, several challenges remain. These include the limited use of real-world data, issues of representativeness, and gaps in accounting for implementation costs, as well as long-term health and non-health benefits. Further research and pilot studies are needed to evaluate both the costs and benefits of PRS applications across diverse populations for multiple health outcomes simultaneously.

## Teaser

This systematic review assesses the economic evaluations of polygenic risk score (PRS)-based interventions across clinical areas. While results are encouraging, evidence mostly relies on hypothetical models. Real-world data and attention to implementation, delivery, and representativeness issues are needed to guide PRS integration into clinical and public health practice.

## Introduction

The increasing emphasis on precision medicine is driving innovation in both research and clinical care.^1^ Advances in gene sequencing technology, coupled with declining costs, have led to a surge in genomic data, making it more accessible and facilitating its integration into routine clinical practice.^2^ However, this integration faces several obstacles, such as shortages of skilled personnel, insufficient information technology infrastructure, and a lack of robust assessment frameworks.^3^ In resource-constrained healthcare systems (HCSs), the evaluation of these technologies is critical in determining their adoption, but it is also highly complex. Traditional assessment methods often struggle with key aspects,^3^ such as keeping pace with the rapid development of new applications; considering their significant impact on patients, families, and society; addressing ethical and legal issues; and accounting for the limited evidence of long-term health and economic outcomes.^4^^,^^5^

Polygenic risk scores (PRSs) are a prime example of a potentially disruptive technology in healthcare.^6^ They are derived from a combination of independent genetic risk variants (e.g., single-nucleotide polymorphisms [SNPs]) linked to a given condition and provide a quantifiable measure of an individual’s genetic predisposition to the disease.^7^ They usually derive from large genome-wide association studies^8^ and are studied across a wide range of healthcare contexts: from prevention, where they can help stratify population risk, to treatment, where, when combined with other clinical risk factors, they may contribute to the identification of high-risk patients who may benefit from specific therapies.^9^ Given the novelty of this approach, the body of evidence on PRSs is continuously evolving, yet their clinical utility is still under discussion.^6^ Indeed, despite the extensive reporting of PRSs in the literature, demonstrating their potential clinical benefits for individual patients or the broader HCS remains challenging.^10^

Within this context, economic evaluations (EEs) are useful because they quantify both the costs and benefits of the alternative strategies that may be applied to a defined population.^11^ A recent review has specifically examined the cost effectiveness of PRSs in the context of cancer screening,^12^ but new data have since emerged.^13^^,^^14^ Furthermore, a growing body of literature is now exploring the effects of introducing PRSs in other settings, such as cardiovascular disease (CVD)^15^^,^^16^^,^^17^^,^^18^^,^^19^ or diabetes,^20^^,^^21^ but no comprehensive synthesis of these findings exists to date. Therefore, we conducted a systematic review of all existing EEs in which a PRS-based approach was modeled (1) to provide an overview of the cost effectiveness across various healthcare contexts, exploring differences and similarities in those contexts, and (2) to critically examine the methodologies employed to quantify both the costs and benefits of introducing this technology into clinical and public health practice. The final aim was to explore the current evidence on PRSs, discuss and summarize the challenges encountered in the evaluation models, and outline directions for future research.

## Methods

This study was performed according to the Cochrane Handbook for Systematic Reviews and the Preferred Reporting Items for Systematic Reviews and Meta-Analyses (PRISMA) statement,^22^^,^^23^ as well as the Center for Reviews and Dissemination guidance on undertaking a systematic review of EEs.^24^ The review protocol was registered at PROSPERO (identifier CRD42023442780). Since this study did not involve primary data collection, institutional review board approval and informed consent were not required.

### Search strategy and study selection

Two reviewers searched the bibliographic databases PubMed, Web of Science, and Scopus using the following search terms: “polygenic risk” AND “economic evaluation.” The string was adapted for each database (Table S1). The search covered reports published from database inception to October 28th, 2024. No restrictions were applied. Duplicate articles were removed, and the title and abstract of all retrieved records were screened. Studies that did not meet the inclusion criteria were excluded. Full texts of potentially relevant articles were examined by two researchers. Disagreements were resolved through discussion, and reasons for exclusion were recorded. The reference lists of retrieved articles were also searched to identify potentially relevant studies.

### Inclusion and exclusion criteria

Eligible articles were EEs quantifying both the costs and benefits of the use of PRSs or any other polygenic risk (PR) stratification strategy in clinical practice, including the consequent healthcare pathways specified. A PRS was defined as “an assessment of the risk of a specific condition based on the collective influence of many genetic variants,” as reported by the National Institutes of Health’s Dictionary of Genetic Terms. As a result, a PRS-based or PR-stratification-based healthcare pathway should consist of the following components: a target population to test, genetic counseling (if applicable), genetic testing to quantify PR, and specific healthcare pathways based on the PRS or PR-stratification result. Adopting this definition, we included original articles that (1) were reported in English or Italian, reflecting the language abilities of the co-authors of this systematic review; (2) had a full EE design (such as cost-effectiveness analysis, cost-utility analysis [CUA], or cost-benefit analysis); and (3) investigated the costs and benefits of the use of PRSs or other PR stratification strategies in any clinical setting and in people of any age. Studies were included regardless of the evaluation perspective. Studies that did not describe an EE, used partial economic design (such as cost analyses, cost-description studies, and cost-outcome descriptions), or did not include any PRS-based or PR-stratification-based healthcare pathways were excluded. Studies that did not refer to any clinical context, were not published in peer-reviewed journals, or were not original articles were also excluded.

### Data collection and quality assessment

For each record included, two reviewers independently extracted the relevant information using a standardized data abstraction form focused on (1) key methodological features, such as type of EE, study perspective, time horizon, currency and baseline year of evaluation, discounting, structure of the model, characteristics and size of the modeled cohort(s), costs and outcomes considered, evidence source of cost and effectiveness data, cost-effectiveness results according to study definitions and results, and sensitivity analyses; and (2) key characteristics of the intervention (disease under study, scope of the PRS testing, target population, PR-based strategy, reference or alternative strategy, and delivery model. Regarding costs, we examined whether PR costs were included in the model, along with other relevant costs, such as those associated with diagnostic tests, treatments, and medical visits, as well as indirect costs, where applicable. As for effectiveness, we analyzed the aspects considered within the defined health states. Additional information, such as authors, journal, funding declaration, and year of publication, was also extracted.

Two independent authors rated the quality of the included EEs using the Quality of Health Economic Studies (QHES) checklist.^25^^,^^26^ The QHES checklist uses a weighted grading system in which the final QHES score ranges from 0 to 100. Articles were considered of high quality if the total score was >75. Details on the calculation of quality scores are provided in the supplemental information. Discrepancies were resolved by consensus.

### Data synthesis

Due to substantial heterogeneity among studies, a meta-analysis was not possible. Articles were grouped according to the disease under study, and three categories were created: cancer, CVD, and other diseases. A narrative synthesis of the identified studies was performed to summarize the key features of the included studies and to compare methods, interventions, and results.

## Results

**Figure 1:**
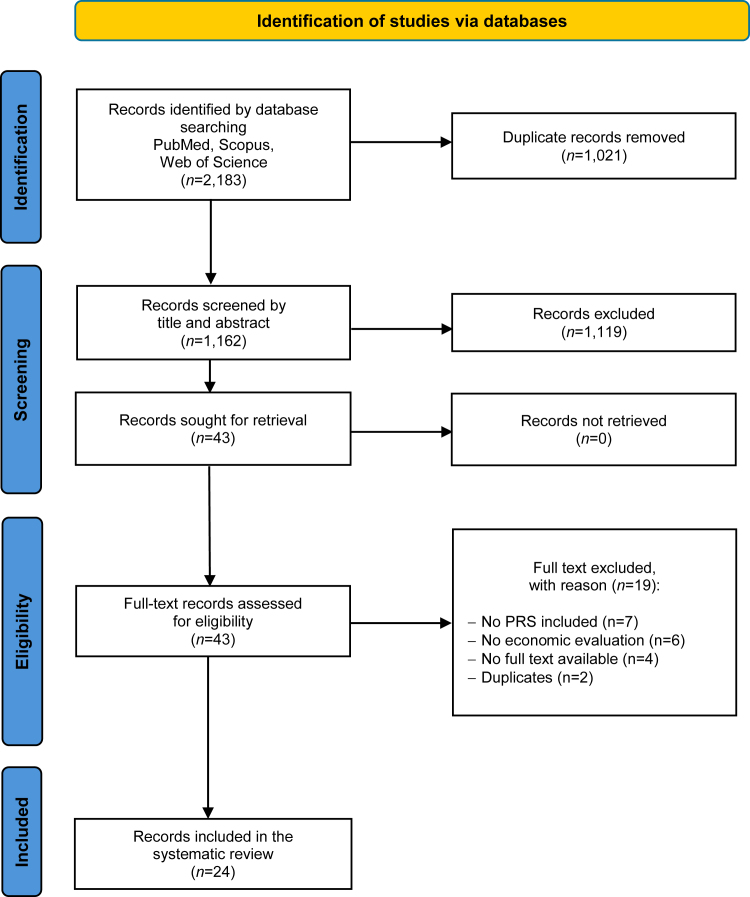
PRISMA flow diagram of the review process PRS, polygenic risk score.

Overall, 2,183 records were identified by database searching (Figure 1). After duplicate removal and screening by title and abstract, 43 articles were selected as eligible for full-text analysis. Of these,19 were excluded, with exclusion reasons recorded, resulting in 24 articles ultimately included in the systematic review.

Of these, 16 studies focused on cancer, specifically prostate cancer (*n* = 6),^27^^,^^28^^,^^29^^,^^30^^,^^31^^,^^32^ colorectal cancer (*n* = 4),^33^^,^^34^^,^^35^^,^^36^ breast cancer (*n* = 3),^13^^,^^37^^,^^38^ lung cancer (*n* = 1),^39^ nasopharyngeal carcinoma (NPC) (*n* = 1),^14^ and multiple cancers simultaneously (*n* = 1), namely lung, liver, gastric, colorectal, esophageal, and female breast cancers.^40^ PRS applications were also evaluated in five studies on CVD,^15^^,^^16^^,^^17^^,^^18^^,^^19^ two studies on type 2 diabetes (T2D),^20^^,^^21^ and one study on primary open-angle glaucoma (POAG).^41^

### General characteristics of the EEs considered by disease

#### Cancer

All EEs were CUAs and were published recently, from 2019^27^ to 2024^13^^,^^14^^,^^18^^,^^19^^,^^36^^,^^39^^,^^40^ (Table 1). The studies were conducted in the United States (*n* = 5),^13^^,^^29^^,^^34^^,^^36^^,^^38^ UK (*n* = 4),^27^^,^^28^^,^^31^^,^^35^ China (*n* = 3),^14^^,^^39^^,^^40^ Sweden (*n* = 2),^30^^,^^32^ Australia (*n* = 1),^33^ and Singapore (*n* = 1).^37^ Almost all studies employed a PRS to inform disease screening^14^^,^^27^^,^^28^^,^^29^^,^^30^^,^^31^^,^^32^^,^^33^^,^^34^^,^^35^^,^^36^^,^^37^^,^^38^^,^^39^^,^^40^ in populations that varied by sex, age, and other risk factors relevant to the specific cancer under consideration. By contrast, only one study used a PRS-based approach to estimate the risk of recurrence and to guide chemotherapy in women with early-stage breast cancer.^13^ When reported (87.5% of studies), the perspective adopted was HCS (*n* = 7),^27^^,^^28^^,^^31^^,^^33^^,^^35^^,^^37^^,^^38^ societal (*n* = 5),^13^^,^^30^^,^^34^^,^^39^^,^^40^ or both (*n* = 2).^32^^,^^36^ The time horizon ranged from up to 79 years of age^14^^,^^39^ to, more commonly, a lifetime.^13^^,^^30^^,^^31^^,^^32^^,^^33^^,^^34^^,^^35^^,^^36^^,^^38^^,^^40^ The baseline year of evaluation ranged from 2014^34^^,^^35^ to 2023.^36^ Discount rates were predominantly 3% for both costs and benefits (*n* = 10),^13^^,^^14^^,^^29^^,^^30^^,^^32^^,^^34^^,^^36^^,^^37^^,^^38^^,^^40^ with other cases reporting 3.5% (*n* = 4)^27^^,^^28^^,^^31^^,^^35^ or 5% (*n* = 2).^33^^,^^39^ Funding sources were disclosed in 14 studies: three reported no funding,^27^^,^^38^^,^^39^ eight received public funding,^14^^,^^29^^,^^31^^,^^33^^,^^34^^,^^35^^,^^36^^,^^40^ one was privately funded,^13^ and two had mixed funding.^30^^,^^32^ The quality of the studies varied from 66/100^37^ to 94/100,^13^^,^^14^^,^^27^^,^^30^^,^^31^^,^^32^^,^^35^^,^^36^^,^^38^ with 11 studies scoring 90 or above.^13^^,^^14^^,^^27^^,^^28^^,^^29^^,^^30^^,^^31^^,^^32^^,^^35^^,^^36^^,^^38^

**Table 1:** General characteristics of the economic evaluations included in the systematic review, by disease

**First author, year**	**Country**	**Type(s) of economic evaluation**	**Target population**	**PRS scope**	**Perspective**	**Time horizon**	**Currency, baseline year of evaluation**	**Discounting (costs, benefits) (%)**	**Study funding**	**Quality**^a^
**Prostate cancer**										

Callender, 2019^27^	UK	CUA	men aged 55–69 years	to inform disease screening	HCS	up to the age of 90 years	pounds (£), 2016	3.5, 3.5	none	94/100
Callender, 2021^28^	UK	CUA	men aged 55–69 years	to inform disease screening	HCS	up to the age of 90 years	pounds (£), 2020	3.5, 3.5	NR	91/100
Hendrix, 2021^29^	USA	CUA	men aged 45–69 years	to inform disease screening	NR	60 years	US dollars ($), 2018	3, 3	public	90/100
Karlsson, 2021^30^	Sweden	CUA	men aged 55–69 years	to inform disease screening	societal	lifetime	Euros (€), 2019	3, 3	combination (public, non-profit, private)	94/100
Keeney, 2022^31^	UK	CUA	men aged ≥30 years	to inform disease screening	HCS	lifetime	pounds (£), 2020	3.5, 3.5	public	94/100
Hao, 2022^32^	Sweden	CUA	men aged 55–69 years	to inform disease screening	HCS and societal	lifetime	Euros (€), 2019	3, 3	combination (public, non-profit)	94/100

**Colorectal cancer**										

Cenin, 2020^33^	Australia	CUA	adults aged 40 years	to inform disease screening	HCS	lifetime	Australian dollars ($AUD), 2016	5, 5	public	82/100
Naber, 2020^34^	USA	CUA	adults aged 40–85 years	to inform disease screening	societal (modified)	lifetime	US dollars ($), 2014	3, 3	public	83/100
Thomas, 2021^35^	UK	CUA	adults aged ≥30 years	to inform disease screening	HCS	lifetime	Pounds (£), 2014	3.5, 3.5	public	94/100
Jiang, 2024^36^	USA	CUA	adults aged 30 years	to inform disease screening	HCS and societal (limited)	lifetime	US dollars ($), 2023	3, 3	public	94/100

**Breast cancer**										

Wong, 2021^37^	Singapore	CUA	women aged 35–74 years	to inform disease screening	HCS	40 years	Singapore dollars (SGD), 2019	3, 3	NR	66/100
Mital, 2022^38^	USA	CUA	women aged 40–49 years	to inform disease screening	HCS	lifetime	US dollars ($), 2020	3, 3	none	94/100
Berdunov, 2024^13^	USA	CUA	women with early-stage invasive breast cancer	to estimate risk of recurrence and inform therapy decisions	societal	lifetime	US dollars ($), 2021	3, 3	private	94/100

**Lung cancer**										

Zhao, 2024^39^	China	CUA	current and former smokers aged 50–74 years	to inform disease screening	societal	until death or age 79 years	Chinese yuan (CNY) 2022	5, 5	none	86/100

**Nasopharyngeal carcinoma**										

Yang, 2024^14^	China	CUA	adults from 30 to 69 years old in high-risk endemic areas of China	to inform disease screening	NR	until death or age 79 years	Chinese renminbi (RMB, ¥) 2022	3, 3	public	94/100

**Multiple cancers (lung, liver, gastric, colorectum, esophagus, and female breast)**										

Xia, 2024^40^	China	CUA	population within age ranges for cancer screening in China	to inform disease screening	societal	lifetime	US dollars ($) 2022	3, 3	public	86/100

**Cardiovascular diseases**										

Kiflen, 2022^15^	Canada	CUA	adults aged 40–69 years with intermediate CVD risk	to inform preventive therapy for CVDs	HCS	10 years	Canadian dollars (CAD), 2006	1.5, 1.5	NR	100/100
Mujwara, 2022^16^	USA	CUA	adults aged 40 years with borderline or intermediate risk of CVD in 10 years	to inform preventive therapy for CVDs	payer	5 and 10 years	US dollars ($), 2019	3, 3	private	94/100
Mujwara, 2023^17^	USA	CUA	employees with a mean age of 50 years, without pre-existing cardiovascular conditions	to inform prevention programs for CVDs, including preventive therapy	self-insured employer	5 years	US dollars ($), 2019	3, 3	NR	91/100
Kelemen, 2024^19^	UK	CUA	men aged ≥60 years and women aged ≥65 years	to inform disease screening for AAA	HCS	lifetime	Pounds (£), NR	NR	public	87/100
Vernon, 2024^18^	Australia	CUA	Australian population aged ≥20 years	to inform prevention programs for CVDs, including preventive therapy	NR	10 years	Australian dollars ($AUD), 2011, 2020	NR	public	62/100

**Type 2 diabetes**										

Guinan, 2021^20^	Canada	CUA	patients with T2D	to inform prevention programs for DN, including preventive therapy	HCS and societal	5 years	Canadian dollars (CAD), 2019	1.5, 1.5	combination (public, private)	87/100
Martikainen, 2022^21^	Finland	CUA	adults aged 30–79 years with high risk for T2D in 10 years	to inform prevention programs for T2D, including preventive therapy	societal	lifetime	Euros (€), 2017	3, 3	public	94/100

**Primary open-angle glaucoma**										

Liu, 2022^41^	UK, Australia	CUA	adults ≥40 years	to inform disease screening	payer	lifetime	Australian dollars ($AUD), 2019; pounds (£), 2019	5, 5	public	94/100

AAA, abdominal aortic aneurism; CUA, cost-utility analysis; CVDs, cardiovascular diseases; DN, diabetic nephropathy; HCS, healthcare system; NR, not reported; PRS, polygenic risk score; T2D, type 2 diabetes; UK, United Kingdom; USA, United States of America.

a The calculation of quality scores are provided in the supplemental information.

#### CVD

Studies involving CVD were mainly conducted in North America^15^^,^^16^^,^^17^ and were published from 2022^15^^,^^16^ to 2024^18^^,^^19^ (Table 1). They were all CUAs, of which two evaluations employed a PRS to guide preventive therapy in adults at intermediate risk of CVD,^15^^,^^16^ while two studies^17^^,^^18^ used a PRS to inform CVD primary prevention programs, including eligibility for preventive therapy, and in another case, a PRS was incorporated into screening programs for abdominal aortic aneurism (AAA).^19^ Perspectives were HCS (*n* = 2),^15^^,^^19^ payer or self-insured employer (*n* = 2),^16^^,^^17^ or non-reported (*n* = 1),^18^ with time horizons from 5 to 10 years^15^^,^^16^^,^^17^^,^^18^ and up to a lifetime.^19^ Evaluations had baseline years from 2006^15^ to 2020.^18^ When discounting rates for costs and benefits were reported (*n* = 3), they were either 3%^16^^,^^17^ or 1.5%.^15^ Quality scores ranged from 62/100^18^ to 100/100.^15^ Funding sources included public (*n* = 2),^18^^,^^19^ private (*n* = 1),^16^ and non-reported (*n* = 2).^15^^,^^17^

#### Other diseases

Two CUAs published in 2021^20^ and 2022^21^ evaluated PRS-based interventions to inform prevention programs for diabetic nephropathy (DN) in Canadian patients with T2D^20^ or prevention programs for T2D in Finnish adults at high risk^21^ (Table 1). The perspective was societal^21^ or HCS and societal.^20^ Time horizons ranged from 5 years^20^ to a lifetime.^21^ Evaluations had baseline years in 2017^21^ and 2019^20^ and applied discount rates of 1.5%^20^ or 3%.^21^ Study quality was 87/100^20^ and 94/100,^21^ with funding either public^21^ or mixed.^20^ A single CUA^41^ published in 2022 evaluated a PRS to inform screening for POAG in adults aged 40 years and older in the UK and Australia. The study used a payer perspective with a lifetime horizon. The evaluation had 2019 as the baseline year and applied a 5% discount rate. The study was funded publicly, and quality was 94/100.

### Characteristics of modeled healthcare pathways considered by disease

#### Cancer

The majority of EEs on cancer used either microsimulation (*n* = 8)^29^^,^^30^^,^^31^^,^^32^^,^^33^^,^^34^^,^^35^^,^^38^ or Markov models (*n* = 6)^13^^,^^27^^,^^28^^,^^36^^,^^37^^,^^39^ (Table 2). All analyses relied exclusively on hypothetical cohorts,^13^^,^^14^^,^^27^^,^^28^^,^^29^^,^^30^^,^^31^^,^^32^^,^^33^^,^^34^^,^^35^^,^^36^^,^^37^^,^^38^^,^^39^^,^^40^ whose sizes, when reported (68.8% of studies), ranged from 1,000 individuals^36^ to 100 million individuals.^29^^,^^33^ Ethnicity or ancestry was never considered in the studies on prostate cancer, whereas it was mentioned or addressed in half of the studies on colorectal cancer (*n* = 2),^34^^,^^35^ two studies on breast cancer,^13^^,^^37^ and the study on NPC.^14^ With regard to the healthcare pathways considered, for prostate cancer, PRS-based screening strategies were based either on PR alone^31^ or on a combination of PRS and other factors, especially age and/or prostate-specific antigen (PSA) levels.^27^^,^^28^^,^^29^^,^^30^^,^^32^ Comparator strategies included both no screening and screening based on the other factors only.^27^^,^^28^^,^^29^^,^^30^^,^^31^^,^^32^ Stratified screening strategies for colorectal cancer always incorporated polygenic profiles alongside other factors, while comparator strategies aligned with clinical guidelines, all focusing on age-based screening.^33^^,^^34^^,^^35^^,^^36^ In the two PRS-based stratified screening strategies for breast cancer, they were either based on polygenic profiles alone^38^ or in combination with age,^13^^,^^37^ with comparators including screening based on age only^37^ or no screening and screening based on a combination of factors.^38^ Another study on breast cancer^13^ instead used a PRS to guide chemotherapy, comparing it to chemotherapy guided by clinical and pathological risk factors. The PRS-based strategy for lung cancer^39^ involved stratified low-dose computed tomography (LDCT) screening based on the polygenic profile, while the alternatives included either no screening or LDCT screening based on risk factors other than genetic profile. The single evaluation investigating NPC^14^ focused on Epstein-Barr virus (EBV) serological screening based solely on the polygenic profile, with comparator strategies including both no screening and age-based screening. The study^40^ exploring PRS-based interventions across multiple cancer types investigated a strategy involving screening 25% of the PRS-defined high-risk population, compared to either no screening or screening 25% of the general population. Only two studies on prostate cancer^30^^,^^32^ mentioned the delivery model, specifically the administration of screening through general practitioners.

**Table 2:** General characteristics of the modeled healthcare pathways considered in the economic evaluations included in the systematic review, by disease

**Author, year**	**Structure of the model**	**Cohort description**	**Ethnicity or ancestry considered**	**PRS-based strategy**	**Reference or alternative strategy**	**Delivery model considered**
**Prostate cancer**						

Callender, 2019^27^	Markov	hypothetical, three cohorts of 4.48 million individuals each	no	stratified screening based on polygenic profile and age	• no screening • screening based on age and PSA level	no
Callender, 2021^28^	Markov	hypothetical, three cohorts of 4.48 million individuals each	no	stratified screening based on polygenic profile and age	• no screening • screening based on age	no
Hendrix, 2021^29^	MS	hypothetical, 100 million individuals	no	stratified screening based on polygenic profile and age, with different time intervals	• no screening • screening based on age and PSA level	no
Karlsson, 2021^30^	MS	hypothetical, cohort size NR	no	stratified screening based on reflex S3M test results (that included PRS) and PSA level, with different time intervals	• no screening • screening based on PSA level	screening administered through GPs
Keeney, 2022^31^	MS	hypothetical, 10 million individuals	no	stratified screening based only on polygenic profile with different intervals	• no screening • screening at different ages and with different intervals	no
Hao, 2022^32^	MS	hypothetical, cohort size NR	no	stratified screening based on reflex S3M test results (that included PRS) and PSA level, with different time intervals	• no screening • MRI based on PSA and TBx/SBx	screening administered through GPs

**Colorectal cancer**						

Cenin, 2020^33^	MS	hypothetical, 100 million individuals	no	stratified screening based on polygenic profile and family history	screening based on age	no
Naber, 2020^34^	MS	hypothetical, cohort size NR	discussion on how much adherence might differ by ethnicity	stratified screening based on polygenic profile, age, and number of colonoscopies, with different time intervals	screening based on age	no
Thomas, 2021^35^	MS	hypothetical, cohort size NR	ethnicity was considered as a phenotypic risk factor	stratified screening based on polygenic profile, BMI, alcohol consumption, smoking, physical activity, and ethnicity	screening based on age	no
Jiang, 2024^36^	decision tree and Markov	hypothetical, 1,000 individuals	no	stratified screening based on polygenic profile and LS status	screening based on age and family history	no

**Breast cancer**						

Wong, 2021^37^	Markov	hypothetical, cohort size NR	risk group percentiles were adjusted to account for Asian ancestry	stratified screening based on polygenic profile and age	screening based on age	no
Mital, 2022^38^	hybrid decision tree and MS	hypothetical, 100,000 individuals	no	stratified screening based only on polygenic profile	• no screening • screening based on different combinations of age, family history, and AI results	no
Berdunov, 2024^13^	Markov	hypothetical, 14,800 individuals	discussion on how gene assay’s effectiveness and cost effectiveness vary by ethnicity, requiring further study	use of adjuvant chemotherapy guided by polygenic profile	use of adjuvant chemotherapy guided by clinical and pathological risk factors	no

**Lung cancer**						

Zhao, 2024^39^	Markov	hypothetical, three cohorts of 10,000 individuals each	no	stratified LDCT screening based on polygenic profile	• no screening • LDCT screening based on other risk factors	no

**Nasopharyngeal carcinoma**						

Yang, 2024^14^	Markov	hypothetical, 100,000 individuals	the study highlights ethnicity impact on NPC incidence but does not address cost-effectiveness variations across ethnicities	stratified screening based only on polygenic profile	• no screening • screening based on age	no

**Multiple cancers (lung, liver, gastric, colorectum, esophagus, and female breast)**						

Xia, 2024^40^	NCC mathematical modeling framework	hypothetical, 22,519,389 individuals	no	screening 25% of PRS-defined high-risk population	• no screening • screening 25% of the general population	no

**Cardiovascular diseases**						

Kiflen, 2022^15^	Markov	real-world cohort, 96,116 individuals	discussion on PRS performance and predictive differences across ethnicities	statin therapy eligibility based on polygenic profile and guidelines	statin therapy eligibility based on guidelines	no
Mujwara, 2022^16^	Markov	hypothetical, 47,108 individuals	mention of the cohort’s initial distribution, derived from a large multi-center, multi-ancestry study	statin therapy eligibility based on CAD-PRS and PCE	statin therapy eligibility based on PCE	no
Mujwara, 2023^17^	Markov	hypothetical, 47,108 individuals	mention of the cohort’s initial distribution, derived from a large multi-center, multi-ancestry study	stratified CVD prevention program based on CAD-PRS and traditional risk factors	• no workplace prevention program • standard CVD workplace program based on traditional risk factors	employee self-administration of genetic test
Kelemen, 2024^19^	discrete event simulation model	real-world cohort, 1 million individuals	restriction to individuals of European ancestry	stratified screening for AAA based on polygenic profile and smoking status	screening strategy for AAA based on sex and age	no
Vernon, 2024^18^	system dynamics model	hypothetical, entire Australian population	no	stratified CVD prevention program based on CAD-PRS and traditional risk factors	stratified CVD prevention program based on traditional risk factors	PRS included into primary prevention within HHC

**Type 2 diabetes**						

Guinan, 2021^20^	Markov	real-world cohort, 4,098 individuals	PRS adjusted for ethnicity, among the other variables	stratified therapy for DN prevention based only on polygenic profile	DN screening based on albuminuria detection and GFR decline	no
Martikainen, 2022^21^	MS	real-world cohort, 313,000 individuals	discussion on how PRS may lack transferability and be limited to the Finnish population	stratified T2D prevention program based on polygenic profile, age, sex, and FINDRISC	stratified T2D prevention program based on age, sex, and FINDRISC	no

**Primary open-angle glaucoma**						

Liu, 2022^41^	Markov	real-world cohort, 11,782,538 Australians and 33,618,730 Britons	no	optometrist and/or ophthalmologist screening based on polygenic profile and age	conventional POAG care pathway based on incidental or symptomatic detection	no

AAA, abdominal aortic aneurysm; AI, artificial intelligence; BMI, body mass index; CAD-PRS, polygenic risk score for coronary artery disease; CVDs, cardiovascular diseases; DN, diabetic nephropathy; FINDRISC, Finnish diabetes risk score; GFR, glomerular filtration rate; GWAS, genome-wide association study; HHC, heart health check; LDCT, low-dose computed tomography; LS, Lynch syndrome; MRI, magnetic resonance imaging; MS, microsimulation; NCC, National Cancer Center; NPC, nasopharyngeal carcinoma; NR, not reported; PCE, pooled cohort equation; POAG, primary open-angle glaucoma; PRS, polygenic risk score; PSA, prostate-specific antigen; SBx, transurethral ultrasound-guided systematic biopsy; S3M, Stockholm-3 model; SNP, single-nucleotide polymorphism; TBx, MRI-guided targeted biopsy; T2D, type 2 diabetes; UK, United Kingdom.

#### CVD

The five evaluations^15^^,^^16^^,^^17^^,^^18^^,^^19^ that examined PRS-based interventions for CVD primarily employed Markov models^15^^,^^16^^,^^17^ (Table 2). All but two studies^15^^,^^19^ used hypothetical cohorts. Cohort sizes ranged from slightly over 40,000 individuals^17^ to the entire Australian population.^18^ Most studies^15^^,^^16^^,^^17^^,^^19^ addressed or mentioned ethnicity in their analyses. PRS-based strategies used different approaches, including (1) eligibility for preventive statin therapy based on polygenic profile and guidelines^15^; (2) eligibility for preventive statin therapy based on a combination of coronary artery disease-PRS (CAD-PRS) and pooled cohort equation (PCE) risk^16^; (3) stratified CVD prevention programs, including eligibility for preventive statin therapy based on a PRS and traditional risk factors^17^; (4) a stratified screening program for AAA by ultrasound scanning based on PRS and smoking status^19^; and (5) CVD primary prevention strategies, including eligibility for preventive statin therapy, based on CAD-PRS and traditional risk factors.^18^ Comparator strategies were eligibility for preventive statin therapy based solely on guidelines,^15^ eligibility for preventive statin therapy based only on PCE,^16^ no CVD prevention program or a standard CVD program,^17^ AAA screening based on age and sex,^19^ and CVD primary prevention programs based on traditional risk factors.^18^ Only two studies addressed how the intervention was delivered: one^17^ in which employees self-administered the genetic test and the other^18^ where the PRS was incorporated into primary prevention data.

#### Other diseases

Two evaluations investigated PRS-based interventions for T2D using Markov^20^ or microsimulation models^21^ (Table 2). Both studies used real-world cohorts, comprising approximately 4,000^20^ and 300,000 individuals,^21^ respectively. Ethnicity was always considered. The PRS-based strategies involved stratified DN prevention therapy based solely on the polygenic profile^20^ and a stratified T2D prevention program based on a combination of polygenic profile and other risk factors.^21^ Comparator strategies included annual DN screening based on clinical features^20^ and a stratified T2D prevention program using risk factors other than the genetic profile.^21^ Delivery strategies were not addressed in either study. One study^41^ on POAG employed a Markov model and a real-world cohort of approximately 11 million Australians and 33 million Britons, with no mention of ethnicity. The two strategies compared were (1) screening by optometrist and/or ophthalmologist based on polygenic profile and age and (2) a conventional pathway based on incidental or symptomatic detection. The delivery model was not mentioned.

### Healthcare costs considered by disease

#### Cancer

All evaluations except two^35^^,^^37^ included PRS costs in their analyses, although one study did not report it^13^ (Table 3). When reported, PRS costs were mainly estimated from commercially available tests (*n* = 6)^13^^,^^29^^,^^32^^,^^33^^,^^34^^,^^38^ or were drawn from the literature (*n* = 3).^14^^,^^31^^,^^36^ In all applicable cases (*n* = 15), diagnostic test costs (i.e., those associated with the diagnosis of the cancer under study) were consistently considered,^14^^,^^27^^,^^28^^,^^29^^,^^30^^,^^31^^,^^32^^,^^33^^,^^34^^,^^35^^,^^36^^,^^37^^,^^38^^,^^39^^,^^40^ while treatment costs were included in all studies.^13^^,^^14^^,^^27^^,^^28^^,^^29^^,^^30^^,^^31^^,^^32^^,^^33^^,^^34^^,^^35^^,^^36^^,^^37^^,^^38^^,^^39^^,^^40^ Medical visit costs were included in half of the studies on prostate cancer,^27^^,^^29^^,^^31^ one study on breast cancer,^38^ the study on NPC,^14^ and the study on multiple cancers.^40^ Costs were predominantly derived from the literature alone (*n* = 5)^27^^,^^28^^,^^35^^,^^36^^,^^37^ or from a combination of the literature and other sources (*n* = 8).^13^^,^^14^^,^^29^^,^^31^^,^^32^^,^^33^^,^^34^^,^^38^ Indirect costs were considered in approximately half of the studies (*n* = 7), with three evaluations employing a human capital approach^30^^,^^32^^,^^40^ and the others relying on the literature or survey data.^13^^,^^34^^,^^36^^,^^39^

**Table 3:** Healthcare costs considered in the economic evaluations included in the systematic review, by disease

**Author, year**	**Costs**							
	**PRS costs**	**Source PRS costs**	**Diagnostic test costs**	**Treatment costs**	**Medical visits**	**Health cost sources**	**Indirect costs**^a^	**Source of indirect costs**^a^
**Prostate cancer**								

Callender, 2019^27^	yes (PR stratification: £25)	laboratory costs	yes	yes	yes	literature	N/A	N/A
Callender, 2021^28^	yes (PR stratification: £50)	personal communication of tariffs	yes	yes	–	literature	N/A	N/A
Hendrix, 2021^29^	yes (genomic risk test: $250)	commercially available test	yes	yes	yes	institutional sources, literature	–	N/A
Karlsson, 2021^30^	yes (S3M test: €196)	NR	yes	yes	–	institutional sources	yes	human capital approach
Keeney, 2022^31^	yes (PR stratification: £25)	literature	yes	yes	yes	literature, guidelines	N/A	N/A
Hao, 2022^32^	yes (S3M test: €217)	commercially available test	yes	yes	–	institutional sources, literature, guidelines	yes	human capital approach

**Colorectal cancer**								

Cenin, 2020^33^	yes (polygenic test: $200)	commercially available test	yes	yes	–	institutional sources, literature	N/A	N/A
Naber, 2020^34^	yes (polygenic test: $200)	commercially available test	yes	yes	–	institutional sources, literature	yes	personal communication, literature
Thomas, 2021^35^	–	N/A	yes	yes	–	literature	N/A	N/A
Jiang, 2024^36^	yes ($250 per LS + PRS genomic screening)	assumption, literature	yes	yes	–	literature	yes	literature

**Breast cancer**								

Wong, 2021^37^	–	N/A	yes	yes	–	literature	N/A	N/A
Mital, 2022^38^	yes (OncoArray genetic test: $115)	commercially available test	yes	yes	yes	institutional sources, literature	N/A	N/A
Berdunov, 2024^13^	yes (price: NR)	commercially available test	N/A^b^	yes	–	literature, assumption, expert evaluation	yes	literature

**Lung cancer**								

Zhao, 2024^39^	yes (PRS screening cost: 280 CNY)	survey data	yes	yes	–	survey data	yes	survey data

**Nasopharyngeal carcinoma**								

Yang, 2024^14^	yes (PRS cost: ¥120.00)	literature	yes	yes	yes	literature, expert evaluation	–	N/A

**Multiple cancers (lung, liver, gastric, colorectum, esophagus, and female breast)**								

Xia, 2024^40^	yes (PRS stratification: $100)	assumption	yes	yes	yes	pilot cancer screening	yes	human capital approach

**Cardiovascular diseases**								

Kiflen, 2022^15^	yes (genotyping PRS: $70)	assumption	N/A^c^	yes	–	institutional sources, literature	N/A	N/A
Mujwara, 2022^16^	yes (PRS test: $100)	commercially available test	N/A^c^	yes	yes	literature	N/A	N/A
Mujwara, 2023^17^	yes (CAD-PRS test: $145)	commercially available test	N/A^c^	yes	yes	literature	yes	literature, assumption
Kelemen, 2024^19^	yes (PRS profiling: $0)	assumption	yes	yes	–	literature	N/A	N/A
Vernon, 2024^18^	yes (PRS test: A$147.20)	literature	N/A^c^	yes	yes	institutional sources	N/A	N/A

**Type 2 diabetes**								

Guinan, 2021^20^	yes (PRS test: $400)	commercially available test	yes	yes	–	institutional sources, literature	yes	government/institutional sources, literature
Martikainen, 2022^21^	yes (PRS test: €50)	assumption	yes	yes	–	institutional sources, literature	yes	literature

**Primary open-angle glaucoma**								

Liu, 2022^41^	yes (genetic screening test: $350 AU, £175 UK)	commercially available test, literature	yes	yes	–	institutional sources, literature	N/A	N/A

LS, Lynch syndrome; N/A, not applicable; NR, not reported; PR, polygenic risk; PRS, polygenic risk score; S3M, Stockholm-3 model; –, no data.

a Indirect costs are applicable only when a societal perspective is adopted.

b This study modeled PRS to guide chemotherapy.

c These studies included diagnostic costs as part of the treatment costs when modeling acute CVD events.

#### CVD

For CVD, all five studies incorporated PRS costs derived from assumptions,^15^^,^^19^ commercially available tests,^16^^,^^17^ and the literature^18^ (Table 3). However, one study assumed that generating a PRS profile in the future would not incur additional costs.^19^ Costs for disease diagnosis were considered applicable in the only study that included it.^19^ Treatment costs of CVD events were universally included, while medical visit costs were included in three studies.^16^^,^^17^^,^^18^ Costs were derived from the literature (*n* = 3)^16^^,^^17^^,^^19^ and/or institutional sources (*n* = 2).^15^^,^^18^ Only one study incorporated indirect costs, drawing on existing literature and assumptions.^17^

#### Other diseases

For T2D, both studies incorporated PRS costs, which were derived from commercially available tests^20^ and assumptions,^21^ while the single study on POAG^41^ sourced PRS costs from commercially available tests and the literature (Table 3). Diagnostic costs were always considered,^20^^,^^21^^,^^41^ as were treatment costs,^20^^,^^21^^,^^41^ while medical visits were always omitted. Costs were always estimated based on institutional sources and the literature.^20^^,^^21^^,^^41^ Only the two studies on T2D accounted for indirect costs, drawing on data from institutional sources^20^ and the literature.^21^

### Benefit and cost-effectiveness outcomes considered by disease

#### Cancer

The EEs on cancer mainly used as health states only cancer development and death (68.8%),^13^^,^^14^^,^^27^^,^^28^^,^^30^^,^^35^^,^^36^^,^^37^^,^^38^^,^^39^ while three studies also added treatment^29^^,^^31^^,^^32^^,^^34^ and two studies also included diagnosis^31^^,^^32^^,^^34^ (Table 4). Utility values were derived predominantly from previous studies (*n* = 9)^13^^,^^14^^,^^28^^,^^30^^,^^32^^,^^33^^,^^36^^,^^37^^,^^38^ or surveys (*n* = 5),^27^^,^^31^^,^^35^^,^^39^^,^^40^ with two evaluations also including assumptions.^29^^,^^34^ Prostate cancer screening showed overall positive results after including PRSs in the healthcare pathways, with four studies concluding that PRS-based strategies were cost effective.^27^^,^^28^^,^^30^^,^^32^ However, one study indicated that PR-stratified strategies improved outcomes only for a subset of participants,^29^ while another study reported that PRS-based screening was not cost effective.^31^ With colorectal cancer, the results were mixed: one study found PRS-based approaches to be cost effective compared to the alternatives,^35^ two studies documented that the comparators were more convenient,^33^^,^^34^ and the last study observed marginal cost effectiveness for a PRS-based strategy at the population level.^36^ Similarly, PR stratification in breast cancer screening produced contrasting results on cost effectiveness. One study reported favorable outcomes,^37^ while another highlighted the inferiority of PRS-based strategies compared to an alternative that employed artificial intelligence only.^38^ Notably, the only study in which a PRS was employed to guide therapy found that PRS-informed adjuvant chemotherapy decisions were cost effective.^13^ PRS-stratified LDCT screening for lung cancer was found to lack cost effectiveness compared to LDCT alone,^39^ while the CUAs on PRS-based screening for NPC^14^ found improved cost effectiveness for specific participant groups only. In the study that explored screening for multiple cancers simultaneously,^40^ PR-stratified screening appeared to be modestly cost effective. Results were generally tested in complex sensitivity analyses: nine studies^13^^,^^14^^,^^27^^,^^30^^,^^32^^,^^35^^,^^36^^,^^38^^,^^40^ used probabilistic methods, one study used only scenario analyses,^28^ and three studies used both probabilistic and scenario analyses,^31^^,^^37^^,^^39^ whereas just two studies conducted one-way sensitivity analysis alone.^29^^,^^34^

**Table 4:** Benefits and cost-effectiveness outcomes of the economic evaluations included in the systematic review, by disease

**Author, year**	**Effectiveness measures**		**Conclusions on cost-effectiveness according to study definitions and results**	**Sensitivity or scenario analysis**
	**Aspects considered in health states**	**Source of utilities**		
**Prostate cancer**				

Callender, 2019^27^	cancer development and death	survey estimating utility values	age- and PRS-based precision screening appears to be cost effective	probabilistic
Callender, 2021^28^	cancer development and death	previous studies investigating utilities	age- and PRS-based precision screening appears to be cost effective	scenario
Hendrix, 2021^29^	cancer development, treatment, and death	assumption and previous studies investigating utilities	polygenic risk-stratified strategies improved outcomes only for a subset of participants	one-way
Karlsson, 2021^30^	cancer development and death	previous studies investigating utilities	screening with the S3M test was cost effective compared to screening with the PSA test alone	one-way, probabilistic
Keeney, 2022^31^	cancer diagnosis, development, treatment, and death	survey estimating utility values	polygenic risk-stratified screening does not appear to be cost effective	one-way, probabilistic, scenario
Hao, 2022^32^	cancer diagnosis, development, treatment, and death	previous studies investigating utilities	screening with the S3M test was predicted to be cost effective	one-way, probabilistic

**Colorectal cancer**				

Cenin, 2020^33^	not explicitly reported but about cancer diagnosis, development, treatment, and death	previous studies investigating utilities	family history- and PRS-based screening does not appear to be cost effective	one-way, scenario
Naber, 2020^34^	cancer diagnosis, development, treatment, and death	assumption and previous studies investigating utilities	polygenic risk-stratified screening is not cost effective	one-way
Thomas, 2021^35^	cancer development and death	survey estimating utility values	polygenic risk-stratified screening appears to be cost effective	probabilistic
Jiang, 2024^36^	cancer development and death	previous studies investigating utilities	population-level LS + PRS screening is marginally cost effective	one-way, probabilistic

**Breast cancer**				

Wong, 2021^37^	cancer development and death	previous studies investigating utilities	polygenic risk-stratified screening appears to be cost effective	one-way, probabilistic, scenario
Mital, 2022^38^	cancer development and death	previous studies investigating utilities	polygenic risk-stratified screening is not cost effective compared with AI-stratified screening	one-way, probabilistic
Berdunov, 2024^13^	cancer development and death	previous studies investigating utilities	adjuvant chemotherapy decisions based on the recurrence score appear to be cost effective	one-way, probabilistic

**Lung cancer**				

Zhao, 2024^39^	cancer development and death	survey estimating utility values	polygenic risk-stratified LDCT screening is not cost effective compared with LDCT-only screening	one-way, probabilistic, scenario

**Nasopharyngeal carcinoma**				

Yang, 2024^14^	cancer development and death	previous studies investigating utilities	polygenic risk-stratified screening improves the cost effectiveness only among a subset of participants	one-way, probabilistic

**Multiple cancers (lung, liver, gastric, colorectum, esophagus, and female breast)**				

Xia, 2024^40^	not explicitly reported, but about cancer development and death	survey estimating utility values	polygenic risk-stratified screening appears to be modestly cost effective	one-way, probabilistic

**Cardiovascular diseases**				

Kiflen, 2022^15^	cardiovascular disease development and death	survey and previous studies investigating utilities	using PRS alongside existing guidelines might be cost effective for CVD	one-way, probabilistic, scenario
Mujwara, 2022^16^	cardiovascular disease development, treatment, and death	previous studies investigating utilities	PCE + CAD-PRS appears to be cost effective when compared with PCE alone	one-way, probabilistic, scenario
Mujwara, 2023^17^	cardiovascular disease development, treatment, and death	previous studies investigating utilities	polygenic testing in a workplace cardiovascular prevention program appears to be cost effective	one-way, probabilistic, scenario
Kelemen, 2024^19^	cardiovascular disease diagnosis, development, treatment, and death	previous studies investigating utilities	PRS-stratified screening improves the cost effectiveness only among a subset of participants	probabilistic
Vernon, 2024^18^	not explicitly reported but about cardiovascular disease development and death	NR	incorporating a CAD-PRS in a primary prevention setting in Australia is cost effective	NR

**Type 2 diabetes**				

Guinan, 2021^20^	renal disease development and death	previous studies investigating utilities	polygenic risk stratification appears to be cost effective	one-way, probabilistic, scenario
Martikainen, 2022^21^	diabetes development and death	survey estimating utility values	including PRS in the risk estimation appears to be cost effective	one-way, probabilistic, scenario

**Primary open-angle glaucoma**				

Liu, 2022^41^	glaucoma development and death	assumption and previous studies investigating utilities	incorporating a PRS for POAG screening seems a promising cost-effectiveness strategy	one-way, probabilistic, scenario

AI, artificial intelligence; CAD, coronary artery disease; CRC, colorectal cancer; CVD, cardiovascular disease; ESRD, end-stage renal disease; ICER, incremental cost-effectiveness ratio; LDCT, low-dose computed tomography screening; LS, Lynch syndrome; LY, life years; MRI, magnetic resonance imaging; NMB, net monetary benefit; NPC, nasopharyngeal carcinoma; NR, not reported; PCE, pooled cohort equation; POAG, primary open-angle glaucoma; PRS, polygenic risk score; PSA, prostate-specific antigen; QALY, quality-adjusted life years; S3M, Stockholm-3 model; T2D, type 2 diabetes; UK, United Kingdom.

#### CVD

Health states considered in CVD were mainly CVD development, treatment, and death. When reported, utilities were derived mainly from previous studies^16^^,^^17^^,^^19^ (Table 4). Studies on CVD generally found that incorporating a PRS into existing screening programs was cost effective,^15^^,^^16^^,^^17^^,^^18^ with only one study reporting that cost effectiveness was restricted to a specific subset of participants.^19^ One study did not report information on sensitivity analysis,^18^ while the others included complex analyses.^15^^,^^16^^,^^17^^,^^19^

#### Other diseases

For T2D, both studies^20^^,^^21^ reported that including a PRS in screening strategies was cost effective, similar to PRS-based screening for POAG, which represented a promising cost-effective intervention^41^ (Table 4). In all cases, findings were supported by extensive sensitivity analyses. As for the health states considered, one evaluation focused on the development of diabetic renal disease,^20^ another centered on diabetes itself,^21^ while the last study^41^ considered glaucoma development and death. Utilities were derived mainly from previous studies^20^^,^^41^ or surveys.^21^

## Discussion

Systematic reviews of EEs enable the identification and assessment of healthcare pathways, supporting the translation of research findings into clinical and public health practice.^42^^,^^43^ This review examined the costs and benefits of introducing PR-based approaches in different scenarios and identified a growing number of recent studies published since 2019, most likely reflecting the increasing interest in PRS implementation. While this interest was initially focused on oncology, it now appears to be rapidly expanding to other clinical conditions, demonstrating the potential applicability of PRSs across a wide range of healthcare settings.^44^ In general, the studies we reviewed aligned with the dual perspective that PRSs can be used to (1) minimize unnecessary interventions in low-risk individuals or (2) maximize health benefits for high-risk population subgroups, objectives made possible through a PR-based stratification and the adjustment of the distribution of individuals across PR strata by varying the cutoff values.^39^ In oncology, this approach has been used to refine existing or hypothetical screening programs, such as those for breast cancer (a disease with a sharply increasing incidence in young women^45^), colorectal cancer (which suffers from low adherence to proposed screening tests^33^), prostate cancer (whose cost effectiveness remains a challenge due to the trade-off between overdiagnosis and mortality^27^), and lung cancer (which, in some countries, is limited to individuals with clinical risk factors^46^). In these analyses, the public funding and HCS perspectives were the most prevalent, suggesting an underlying attempt to evaluate PRSs as a means to enhance the cost effectiveness of public health interventions. Conversely, in the other fields, PRSs were mostly modeled to refine eligibility for preventive therapies, with smaller time horizons and the use of different perspectives. However, in most studies, PRS-based strategies were compared to different alternatives, and PRSs were frequently evaluated in association with other risk factors, using this approach to quantify potential improvements in disease-incidence prediction, even though this analysis may expose the studies to survival bias,^12^ especially when middle-aged cohorts are enrolled and the most severe cases may have already been diagnosed. Furthermore, none of the studies considered PRSs as a way of determining disease aggressiveness, and thus, the models did not account for variations in clinical pathways across diseased individuals, in contrast to emerging evidence suggesting that an unfavorable PRS could also be associated with more aggressive disease development and progression.^47^

The results on cost effectiveness were mixed in some cases but generally indicated a positive trend, with CVD showing the greatest consistency both in terms of the number of studies included and homogeneity in supporting PRS implementation. The analyses were often robust and mostly varied the PRS cost, its discrimination power, and uptake rates. Indeed, most studies assumed universal uptake of PRS testing, while individual resistance—particularly due to privacy concerns^17^—remains a major challenge, along with potentially unequal access to PRSs, both of which could exacerbate health disparities.^48^ This general assumption of 100% adherence is crucial, as it has been demonstrated that reduced uptake rates can substantially diminish overall benefits.^18^^,^^35^ Furthermore, the limited predictiveness of PRSs is an issue, as a significant number of incident cases will always be excluded from PRS-stratified screening programs (because they are classified as low risk) yet will account for the majority of cases, a phenomenon known as Rose’s prevention paradox.^49^^,^^50^ Additionally, individual PRS rankings can vary considerably depending on the construction methods used, leading to rank instability that may shift individuals across clinical decision thresholds despite unchanged risk profiles.^51^ Therefore, it is plausible that, along with decreasing costs, enhancing PRS discrimination power (e.g., by discovering new SNPs, combining it with many other risk factors, or incorporating data from different ethnicities and ancestries) could potentially improve the cost effectiveness of these programs, although some uncertainties remain regarding the clinical significance of the health benefits.^37^ Even with perfect discrimination, PRSs inherently explain only a fraction of disease risk: this brings into question whether further efforts should first focus on establishing whether PRS-based strategies can meaningfully contribute to clinical or public health decision-making.^52^ Indeed, for common complex diseases, PRSs struggle to provide clinically relevant predictions because these conditions often result from the interplay of numerous genetic and environmental factors.^53^ Additionally, for diseases with low prevalence, the clinical benefits are even more debatable.^18^^,^^54^ Even with perfect accuracy, the health gains for some individuals would be averaged across the entire population, which, in the case of low-prevalence diseases, could significantly limit the overall population-level impact of PRS-based interventions.^18^ However, collecting data on long-term outcomes is essential to fully evaluate the real-world impact of PRS integration into clinical practice, as current evidence relies primarily on models.^55^ Interestingly, the overall quality of the studies included was high, suggesting that traditional EE assessment tools may not capture all considerations on PRS implementation.^12^ Indeed, we found a few limitations worth discussing. Although microsimulation models are quite flexible in simulating clinical scenarios and are preferable to Markov models,^56^ relying on hypothetical cohorts, as many studies did,^13^^,^^16^^,^^17^^,^^18^^,^^27^^,^^28^^,^^29^^,^^30^^,^^31^^,^^32^^,^^33^^,^^34^^,^^35^^,^^36^^,^^37^^,^^38^^,^^39^^,^^40^ allows for the integration of data from various sources but also often leads to the homogenization of individual variability,^57^ and results are heavily dependent on the model’s assumptions, limiting the generalizability of the findings.^58^ By contrast, the use of clinical trials incorporating PRSs into clinical practice has the potential to validate these tools in real-world settings.^59^^,^^60^ This lack of integration with clinical trials may limit the ability of the included evaluations to accurately reflect the effectiveness, adoption, and impact of PRSs in HCSs. Furthermore, despite being frequently mentioned in the limitations section, ethnicity and ancestry, which influence predisposition to specific conditions and play a crucial role in the representativeness of PRSs, were seldom addressed.^20^^,^^35^^,^^37^ This omission is relevant, as most available PRSs have the greatest predictive power for individuals of a specific ancestry^12^ and none of the included studies conducted EEs across different ancestry groups, despite the growing scientific effort to enhance PRS applicability across diverse ancestral backgrounds.^61^^,^^62^ Moreover, most studies were conducted in high-income countries, and even with an accurate representation of the target population, as in the Finnish study,^21^ the applicability of the results to other populations is not guaranteed.^63^

Another significant issue was the insufficient attention paid to PRS delivery models. This aspect is critical for ensuring realistic evaluations.^55^ Key organizational factors, such as the method of genomic data acquisition and the reorganization of services (e.g., expanding laboratory capacity, workforce training, developing technical infrastructure for large-scale genetic sequencing, etc.), are fundamental but were largely overlooked.^19^^,^^20^^,^^40^ For this reason, conducting pilot studies is essential to gain a better understanding of how PRSs should be integrated into healthcare contexts and to quantify the implementation costs, which remain largely unexamined in current evaluations and most likely depend on clinical settings, delivery models, and population characteristics.^41^ Likewise, indirect costs, which are vital for a comprehensive assessment of the socioeconomic impact of healthcare innovations—and among the reasons why the societal perspective is recommended in healthcare EEs^64^—were mostly ignored.^13^^,^^17^^,^^20^^,^^21^^,^^30^^,^^32^^,^^34^^,^^36^^,^^39^^,^^40^ As for the health benefits, which are central to ongoing discussions on the clinical significance of PRSs,^10^ the studies reviewed here exclusively employed health states and utilities that reflect the clinical aspects of the diseases while neglecting the unique considerations associated with genetic testing, such as the implications of cascade testing (modeled in only one study^36^) and the return of secondary or incidental findings,^65^ which was never considered. Moreover, none of the studies accounted for the health effects associated with knowing one’s own PRS, such as anxiety, false reassurance from negative results, or behavioral changes in high-risk individuals (e.g., dietary improvements^16^^,^^17^), all of which could influence cost effectiveness. In this regard, while real-world trials are designed and implemented, the inclusion of an impact inventory in the EEs could help in the identification of the health and non-health effects that should be considered in a societal reference case analysis.^66^ Lastly, the use of genetic data to calculate PRSs for multiple outcomes remained underexplored. Only Xia et al.^40^ attempted to model the risk for multiple cancers simultaneously; given the versatility of PRSs, which can be applied to multiple diseases,^67^ this is a promising area for future research.

This study has both strengths and limitations. This systematic review provides a comprehensive overview of existing evidence on the EEs of PRS integration into clinical and public health practice across different settings and may serve as a useful foundation for future research and implementation efforts. The inclusion of studies spanning diverse clinical contexts enabled comparisons of both methods and results, highlighting similarities and differences. Additionally, by exploring and discussing the methodologies used to quantify both costs and benefits, this review provides valuable insights into the current body of literature on PRS-based healthcare pathways. In contrast, several limitations should be acknowledged. Given the considerable heterogeneity in disease types, model structures, and approaches used to calculate PRSs, it was impossible to aggregate quantitative data through a meta-analysis. Furthermore, key cost-effectiveness metrics, such as the incremental cost-effectiveness ratio and willingness-to-pay thresholds, were not discussed. This aligns with the review’s purpose, which was not to establish the cost effectiveness of PRS-based programs but rather to investigate the methodologies applied in these studies.

### Conclusions

While the available evidence suggests a positive trend in the cost-effectiveness of PRS implementation, particularly for CVD, challenges remain, especially the limited availability of real-world data, issues with PRS representativeness, gaps in addressing implementation costs and the need to fully account for the health benefits associated with PRS-based testing. These findings underscore the need for pilot studies and further research into the benefits and costs associated with using PRS whose clinical and public health relevance has been established, as well as the application of PRS strategies to more diverse populations and for multiple outcomes simultaneously.

## Data and code availability

All relevant data are included in the article.

## Acknowledgments

The research leading to these results received funding from the 10.13039/501100000780European Union - NextGenerationEU through the Italian Ministry of University and Research under PNRR - M4C2-I1.3 Project PE_00000019 “HEAL ITALIA” to P.V., CUP B53C22004000006. The views and opinions expressed are those of the authors only and do not necessarily reflect those of the European Union or the European Commission. Neither the European Union nor the European Commission can be held responsible for them.

## Author contributions

Concept and design, L.M.S., V.B., M.R., and A.R.; acquisition, analysis, or interpretation of data, L.M.S., V.B., M.R., A.R., A.S., C.I., J.I., and F.P.; drafting of the manuscript, L.M.S., V.B., M.R., and A.R.; critical review of the manuscript for important intellectual content, all authors; supervision, V.B., A.R., G.M., and P.V.

## Declaration of interests

The authors declare no competing interests.

## Supplemental information

Document S1. Table S1 and supplemental information

Document S2. Article plus supplemental information
