# Supplementary material for: Weighing the evidence on costs and benefits of polygenic risk-based approaches in clinical practice: A systematic review of economic evaluations
Source: Am J Hum Genet. 2025 Jun 12;112(8):1735–53. doi: 10.1016/j.ajhg.2025.05.012 (PMC12414691; doi:10.1016/j.ajhg.2025.05.012)
Supplement: Document S1. Table S1 and supplemental information [file mmc1.pdf]

The American Journal of Human Genetics, Volume *112*

Supplemental information

Weighing the evidence on costs and benefits of polygenic risk-based approaches in clinical practice: A systematic review of economic evaluations

Leonardo Maria Siena, Valentina Baccolini, Marianna Riccio, Annalisa Rosso, Giuseppe Migliara, Antonio Sciurti, Claudia Isonne, Jessica Iera, Francesco Pierri, Carolina Marzuillo, Corrado De Vito, Giuseppe La Torre, and Paolo Villari

SUPPLEMENT

Table S1. Search strategies used in the systematic review.

("PRS" OR "polygenic risk*" OR "polygenic score*" OR "genetic risk score*" OR "genetic score*" OR "GWAS" OR "genome wide association*" OR "genome-wide association*" OR "genome-wide genotype*" OR "genome wide genotype*" OR "Genome-Wide Association Study"[Mesh]) AND ("economic evaluation*" OR "economic analysis" OR "cost-effective" OR "cost-effectiveness" OR "cost-benefit" OR "cost-utility" OR "Costs and Cost Analysis"[Mesh])	PubMed
TITLE-ABS-KEY(("PRS" OR "polygenic risk*" OR "polygenic score*" OR "genetic risk score*" OR "genetic score*" OR "GWAS" OR "genome wide association*" OR "genome-wide association*" OR "genome-wide genotype*" OR "genome wide genotype*") AND ("economic evaluation*" OR "economic analysis" OR "cost-effective" OR "cost-effectiveness" OR "cost-benefit" OR "cost-utility"))	Scopus
TS=(("PRS" OR "polygenic risk*" OR "polygenic score*" OR "genetic risk score*" OR "genetic score*" OR "GWAS" OR "genome wide association*" OR "genome-wide association*" OR "genome-wide genotype*" OR "genome wide genotype*") AND ("economic evaluation*" OR "economic analysis" OR "cost-effective" OR "cost-effectiveness" OR "cost-benefit" OR "cost-utility"))	Web of Science

Quality assessment of the articles included in the systematic review according to the Quality of Health Economic Studies (QHES) instrument:

1 - The Quality of Health Economic Studies (QHES) instrument – *Callender, 2019*

No	Questions	Points	Yes	No
1.	Was the study objective presented in a clear, specific, and measurable manner?	7	x	
2.	Were the perspective of the analysis (societal, third-party payer, etc.) and reasons for its selection stated?	4	x	
3.	Were variable estimates used in the analysis from the best available source (i.e., randomized control trial - best, expert opinion - worst)?	8	x	
4.	If estimates came from a subgroup analysis, were the groups pre- specified at the beginning of the study?	1	x (na)	
5.	Was uncertainty handled by (1) statistical analysis to address random events, (2) sensitivity analysis to cover a range of assumptions?	9	x	
6.	Was incremental analysis performed between alternatives for resources and costs?	6	x	
7.	Was the methodology for data abstraction (including the value of health states and other benefits) stated?	5	x	
8.	Did the analytic horizon allow time for all relevant and important outcomes? Were benefits and costs that went beyond 1 year discounted (3% to 5%) and justification given for the discount rate?	7	x	
9.	Was the measurement of costs appropriate and the methodology for the estimation of quantities and unit costs clearly described?	8	x	
10.	Were the primary outcome measure(s) for the economic evaluation clearly stated and did they include the major short-term, long-term, and negative outcomes?	6		x
11.	Were the health outcomes measures/scales valid and reliable? If previously tested valid and reliable measures were not available, was justification given for the measures/scales used?	7	x	
12.	Were the economic model (including structure), study methods and analysis, and the components of the numerator and denominator displayed in a clear, transparent manner?	8	x	
13.	Were the choice of economic model, main assumptions, and limitations of the study stated and justified?	7	x	
14.	Did the author(s) explicitly discuss direction and magnitude of potential biases?	6	x	
15.	Were the conclusions/recommendations of the study justified and based on the study results?	8	x	
16.	Was there a statement disclosing the source of funding for the study?	3	x	
	**TOTAL POINTS**	**100**	94	

2 - The Quality of Health Economic Studies (QHES) instrument – *Callender, 2021*

No	Questions	Points	Yes	No
1.	Was the study objective presented in a clear, specific, and measurable manner?	7	x	
2.	Were the perspective of the analysis (societal, third-party payer, etc.) and reasons for its selection stated?	4	x	
3.	Were variable estimates used in the analysis from the best available source (i.e., randomized control trial - best, expert opinion - worst)?	8	x	
4.	If estimates came from a subgroup analysis, were the groups pre- specified at the beginning of the study?	1	x (na)	
5.	Was uncertainty handled by (1) statistical analysis to address random events, (2) sensitivity analysis to cover a range of assumptions?	9	x	
6.	Was incremental analysis performed between alternatives for resources and costs?	6	x	
7.	Was the methodology for data abstraction (including the value of health states and other benefits) stated?	5	x	
8.	Did the analytic horizon allow time for all relevant and important outcomes? Were benefits and costs that went beyond 1 year discounted (3% to 5%) and justification given for the discount rate?	7	x	
9.	Was the measurement of costs appropriate and the methodology for the estimation of quantities and unit costs clearly described?	8	x	
10.	Were the primary outcome measure(s) for the economic evaluation clearly stated and did they include the major short-term, long-term, and negative outcomes?	6		x
11.	Were the health outcomes measures/scales valid and reliable? If previously tested valid and reliable measures were not available, was justification given for the measures/scales used?	7	x	
12.	Were the economic model (including structure), study methods and analysis, and the components of the numerator and denominator displayed in a clear, transparent manner?	8	x	
13.	Were the choice of economic model, main assumptions, and limitations of the study stated and justified?	7	x	
14.	Did the author(s) explicitly discuss direction and magnitude of potential biases?	6	x	
15.	Were the conclusions/recommendations of the study justified and based on the study results?	8	x	
16.	Was there a statement disclosing the source of funding for the study?	3		x
	**TOTAL POINTS**	**100**	91	

3 - The Quality of Health Economic Studies (QHES) instrument – *Hendrix, 2021*

No	Questions	Points	Yes	No
1.	Was the study objective presented in a clear, specific, and measurable manner?	7	x	
2.	Were the perspective of the analysis (societal, third-party payer, etc.) and reasons for its selection stated?	4		x
3.	Were variable estimates used in the analysis from the best available source (i.e., randomized control trial - best, expert opinion - worst)?	8	x	
4.	If estimates came from a subgroup analysis, were the groups pre- specified at the beginning of the study?	1	x (na)	
5.	Was uncertainty handled by (1) statistical analysis to address random events, (2) sensitivity analysis to cover a range of assumptions?	9	x	
6.	Was incremental analysis performed between alternatives for resources and costs?	6	x	
7.	Was the methodology for data abstraction (including the value of health states and other benefits) stated?	5	x	
8.	Did the analytic horizon allow time for all relevant and important outcomes? Were benefits and costs that went beyond 1 year discounted (3% to 5%) and justification given for the discount rate?	7	x	
9.	Was the measurement of costs appropriate and the methodology for the estimation of quantities and unit costs clearly described?	8	x	
10.	Were the primary outcome measure(s) for the economic evaluation clearly stated and did they include the major short-term, long-term, and negative outcomes?	6		x
11.	Were the health outcomes measures/scales valid and reliable? If previously tested valid and reliable measures were not available, was justification given for the measures/scales used?	7	x	
12.	Were the economic model (including structure), study methods and analysis, and the components of the numerator and denominator displayed in a clear, transparent manner?	8	x	
13.	Were the choice of economic model, main assumptions, and limitations of the study stated and justified?	7	x	
14.	Did the author(s) explicitly discuss direction and magnitude of potential biases?	6	x	
15.	Were the conclusions/recommendations of the study justified and based on the study results?	8	x	
16.	Was there a statement disclosing the source of funding for the study?	3	x	
	**TOTAL POINTS**	**100**	90	

4 - The Quality of Health Economic Studies (QHES) instrument – *Karlsson, 2021*

No	Questions	Points	Yes	No
1.	Was the study objective presented in a clear, specific, and measurable manner?	7	x	
2.	Were the perspective of the analysis (societal, third-party payer, etc.) and reasons for its selection stated?	4	x	
3.	Were variable estimates used in the analysis from the best available source (i.e., randomized control trial - best, expert opinion - worst)?	8	x	
4.	If estimates came from a subgroup analysis, were the groups pre- specified at the beginning of the study?	1	x (na)	
5.	Was uncertainty handled by (1) statistical analysis to address random events, (2) sensitivity analysis to cover a range of assumptions?	9	x	
6.	Was incremental analysis performed between alternatives for resources and costs?	6	x	
7.	Was the methodology for data abstraction (including the value of health states and other benefits) stated?	5	x	
8.	Did the analytic horizon allow time for all relevant and important outcomes? Were benefits and costs that went beyond 1 year discounted (3% to 5%) and justification given for the discount rate?	7	x	
9.	Was the measurement of costs appropriate and the methodology for the estimation of quantities and unit costs clearly described?	8	x	
10.	Were the primary outcome measure(s) for the economic evaluation clearly stated and did they include the major short-term, long-term, and negative outcomes?	6		x
11.	Were the health outcomes measures/scales valid and reliable? If previously tested valid and reliable measures were not available, was justification given for the measures/scales used?	7	x	
12.	Were the economic model (including structure), study methods and analysis, and the components of the numerator and denominator displayed in a clear, transparent manner?	8	x	
13.	Were the choice of economic model, main assumptions, and limitations of the study stated and justified?	7	x	
14.	Did the author(s) explicitly discuss direction and magnitude of potential biases?	6	x	
15.	Were the conclusions/recommendations of the study justified and based on the study results?	8	x	
16.	Was there a statement disclosing the source of funding for the study?	3	x	
	**TOTAL POINTS**	**100**	94	

5 - The Quality of Health Economic Studies (QHES) instrument – *Keeney, 2022*

No	Questions	Points	Yes	No
1.	Was the study objective presented in a clear, specific, and measurable manner?	7	x	
2.	Were the perspective of the analysis (societal, third-party payer, etc.) and reasons for its selection stated?	4	x	
3.	Were variable estimates used in the analysis from the best available source (i.e., randomized control trial - best, expert opinion - worst)?	8	x	
4.	If estimates came from a subgroup analysis, were the groups pre- specified at the beginning of the study?	1	x (na)	
5.	Was uncertainty handled by (1) statistical analysis to address random events, (2) sensitivity analysis to cover a range of assumptions?	9	x	
6.	Was incremental analysis performed between alternatives for resources and costs?	6	x	
7.	Was the methodology for data abstraction (including the value of health states and other benefits) stated?	5	x	
8.	Did the analytic horizon allow time for all relevant and important outcomes? Were benefits and costs that went beyond 1 year discounted (3% to 5%) and justification given for the discount rate?	7	x	
9.	Was the measurement of costs appropriate and the methodology for the estimation of quantities and unit costs clearly described?	8	x	
10.	Were the primary outcome measure(s) for the economic evaluation clearly stated and did they include the major short-term, long-term, and negative outcomes?	6		x
11.	Were the health outcomes measures/scales valid and reliable? If previously tested valid and reliable measures were not available, was justification given for the measures/scales used?	7	x	
12.	Were the economic model (including structure), study methods and analysis, and the components of the numerator and denominator displayed in a clear, transparent manner?	8	x	
13.	Were the choice of economic model, main assumptions, and limitations of the study stated and justified?	7	x	
14.	Did the author(s) explicitly discuss direction and magnitude of potential biases?	6	x	
15.	Were the conclusions/recommendations of the study justified and based on the study results?	8	x	
16.	Was there a statement disclosing the source of funding for the study?	3	x	
	**TOTAL POINTS**	**100**	94	

6 - The Quality of Health Economic Studies (QHES) instrument – *Hao, 2021*

No	Questions	Points	Yes	No
1.	Was the study objective presented in a clear, specific, and measurable manner?	7	x	
2.	Were the perspective of the analysis (societal, third-party payer, etc.) and reasons for its selection stated?	4	x	
3.	Were variable estimates used in the analysis from the best available source (i.e., randomized control trial - best, expert opinion - worst)?	8	x	
4.	If estimates came from a subgroup analysis, were the groups pre- specified at the beginning of the study?	1	x (na)	
5.	Was uncertainty handled by (1) statistical analysis to address random events, (2) sensitivity analysis to cover a range of assumptions?	9	x	
6.	Was incremental analysis performed between alternatives for resources and costs?	6	x	
7.	Was the methodology for data abstraction (including the value of health states and other benefits) stated?	5	x	
8.	Did the analytic horizon allow time for all relevant and important outcomes? Were benefits and costs that went beyond 1 year discounted (3% to 5%) and justification given for the discount rate?	7	x	
9.	Was the measurement of costs appropriate and the methodology for the estimation of quantities and unit costs clearly described?	8	x	
10.	Were the primary outcome measure(s) for the economic evaluation clearly stated and did they include the major short-term, long-term, and negative outcomes?	6		x
11.	Were the health outcomes measures/scales valid and reliable? If previously tested valid and reliable measures were not available, was justification given for the measures/scales used?	7	x	
12.	Were the economic model (including structure), study methods and analysis, and the components of the numerator and denominator displayed in a clear, transparent manner?	8	x	
13.	Were the choice of economic model, main assumptions, and limitations of the study stated and justified?	7	x	
14.	Did the author(s) explicitly discuss direction and magnitude of potential biases?	6	x	
15.	Were the conclusions/recommendations of the study justified and based on the study results?	8	x	
16.	Was there a statement disclosing the source of funding for the study?	3	x	
	**TOTAL POINTS**	**100**	94	

7 - The Quality of Health Economic Studies (QHES) instrument – *Cenin, 2020*

No	Questions	Points	Yes	No
1.	Was the study objective presented in a clear, specific, and measurable manner?	7	x	
2.	Were the perspective of the analysis (societal, third-party payer, etc.) and reasons for its selection stated?	4	x	
3.	Were variable estimates used in the analysis from the best available source (i.e., randomized control trial - best, expert opinion - worst)?	8	x	
4.	If estimates came from a subgroup analysis, were the groups pre- specified at the beginning of the study?	1	x (na)	
5.	Was uncertainty handled by (1) statistical analysis to address random events, (2) sensitivity analysis to cover a range of assumptions?	9	x	
6.	Was incremental analysis performed between alternatives for resources and costs?	6	x	
7.	Was the methodology for data abstraction (including the value of health states and other benefits) stated?	5		x
8.	Did the analytic horizon allow time for all relevant and important outcomes? Were benefits and costs that went beyond 1 year discounted (3% to 5%) and justification given for the discount rate?	7	x	
9.	Was the measurement of costs appropriate and the methodology for the estimation of quantities and unit costs clearly described?	8	x	
10.	Were the primary outcome measure(s) for the economic evaluation clearly stated and did they include the major short-term, long-term, and negative outcomes?	6		x
11.	Were the health outcomes measures/scales valid and reliable? If previously tested valid and reliable measures were not available, was justification given for the measures/scales used?	7		x
12.	Were the economic model (including structure), study methods and analysis, and the components of the numerator and denominator displayed in a clear, transparent manner?	8	x	
13.	Were the choice of economic model, main assumptions, and limitations of the study stated and justified?	7	x	
14.	Did the author(s) explicitly discuss direction and magnitude of potential biases?	6	x	
15.	Were the conclusions/recommendations of the study justified and based on the study results?	8	x	
16.	Was there a statement disclosing the source of funding for the study?	3	x	
	**TOTAL POINTS**	**100**	82	

8 - The Quality of Health Economic Studies (QHES) instrument – *Naber, 2020*

No	Questions	Points	Yes	No
1.	Was the study objective presented in a clear, specific, and measurable manner?	7	x	
2.	Were the perspective of the analysis (societal, third-party payer, etc.) and reasons for its selection stated?	4	x	
3.	Were variable estimates used in the analysis from the best available source (i.e., randomized control trial - best, expert opinion - worst)?	8	x	
4.	If estimates came from a subgroup analysis, were the groups pre- specified at the beginning of the study?	1	x	
5.	Was uncertainty handled by (1) statistical analysis to address random events, (2) sensitivity analysis to cover a range of assumptions?	9	x	
6.	Was incremental analysis performed between alternatives for resources and costs?	6		x
7.	Was the methodology for data abstraction (including the value of health states and other benefits) stated?	5		x
8.	Did the analytic horizon allow time for all relevant and important outcomes? Were benefits and costs that went beyond 1 year discounted (3% to 5%) and justification given for the discount rate?	7	x	
9.	Was the measurement of costs appropriate and the methodology for the estimation of quantities and unit costs clearly described?	8	x	
10.	Were the primary outcome measure(s) for the economic evaluation clearly stated and did they include the major short-term, long-term, and negative outcomes?	6		x
11.	Were the health outcomes measures/scales valid and reliable? If previously tested valid and reliable measures were not available, was justification given for the measures/scales used?	7	x	
12.	Were the economic model (including structure), study methods and analysis, and the components of the numerator and denominator displayed in a clear, transparent manner?	8	x	
13.	Were the choice of economic model, main assumptions, and limitations of the study stated and justified?	7	x	
14.	Did the author(s) explicitly discuss direction and magnitude of potential biases?	6	x	
15.	Were the conclusions/recommendations of the study justified and based on the study results?	8	x	
16.	Was there a statement disclosing the source of funding for the study?	3	x	
	**TOTAL POINTS**	**100**	83	

9 - The Quality of Health Economic Studies (QHES) instrument – *Thomas, 2021*

No	Questions	Points	Yes	No
1.	Was the study objective presented in a clear, specific, and measurable manner?	7	x	
2.	Were the perspective of the analysis (societal, third-party payer, etc.) and reasons for its selection stated?	4	x	
3.	Were variable estimates used in the analysis from the best available source (i.e., randomized control trial - best, expert opinion - worst)?	8	x	
4.	If estimates came from a subgroup analysis, were the groups pre- specified at the beginning of the study?	1	x (na)	
5.	Was uncertainty handled by (1) statistical analysis to address random events, (2) sensitivity analysis to cover a range of assumptions?	9	x	
6.	Was incremental analysis performed between alternatives for resources and costs?	6	x	
7.	Was the methodology for data abstraction (including the value of health states and other benefits) stated?	5	x	
8.	Did the analytic horizon allow time for all relevant and important outcomes? Were benefits and costs that went beyond 1 year discounted (3% to 5%) and justification given for the discount rate?	7	x	
9.	Was the measurement of costs appropriate and the methodology for the estimation of quantities and unit costs clearly described?	8	x	
10.	Were the primary outcome measure(s) for the economic evaluation clearly stated and did they include the major short-term, long-term, and negative outcomes?	6		x
11.	Were the health outcomes measures/scales valid and reliable? If previously tested valid and reliable measures were not available, was justification given for the measures/scales used?	7	x	
12.	Were the economic model (including structure), study methods and analysis, and the components of the numerator and denominator displayed in a clear, transparent manner?	8	x	
13.	Were the choice of economic model, main assumptions, and limitations of the study stated and justified?	7	x	
14.	Did the author(s) explicitly discuss direction and magnitude of potential biases?	6	x	
15.	Were the conclusions/recommendations of the study justified and based on the study results?	8	x	
16.	Was there a statement disclosing the source of funding for the study?	3	x	
	**TOTAL POINTS**	**100**	94	

10 - The Quality of Health Economic Studies (QHES) instrument – *Wong, 2021*

No	Questions	Points	Yes	No
1.	Was the study objective presented in a clear, specific, and measurable manner?	7	x	
2.	Were the perspective of the analysis (societal, third-party payer, etc.) and reasons for its selection stated?	4	x	
3.	Were variable estimates used in the analysis from the best available source (i.e., randomized control trial - best, expert opinion - worst)?	8	x	
4.	If estimates came from a subgroup analysis, were the groups pre- specified at the beginning of the study?	1	x (na)	
5.	Was uncertainty handled by (1) statistical analysis to address random events, (2) sensitivity analysis to cover a range of assumptions?	9	x	
6.	Was incremental analysis performed between alternatives for resources and costs?	6	x	
7.	Was the methodology for data abstraction (including the value of health states and other benefits) stated?	5		x
8.	Did the analytic horizon allow time for all relevant and important outcomes? Were benefits and costs that went beyond 1 year discounted (3% to 5%) and justification given for the discount rate?	7	x	
9.	Was the measurement of costs appropriate and the methodology for the estimation of quantities and unit costs clearly described?	8		x
10.	Were the primary outcome measure(s) for the economic evaluation clearly stated and did they include the major short-term, long-term, and negative outcomes?	6		x
11.	Were the health outcomes measures/scales valid and reliable? If previously tested valid and reliable measures were not available, was justification given for the measures/scales used?	7	x	
12.	Were the economic model (including structure), study methods and analysis, and the components of the numerator and denominator displayed in a clear, transparent manner?	8		x
13.	Were the choice of economic model, main assumptions, and limitations of the study stated and justified?	7		x
14.	Did the author(s) explicitly discuss direction and magnitude of potential biases?	6	x	
15.	Were the conclusions/recommendations of the study justified and based on the study results?	8	x	
16.	Was there a statement disclosing the source of funding for the study?	3	x	
	**TOTAL POINTS**	**100**	66	

11 - The Quality of Health Economic Studies (QHES) instrument – *Mital, 2022*

No	Questions	Points	Yes	No
1.	Was the study objective presented in a clear, specific, and measurable manner?	7	x	
2.	Were the perspective of the analysis (societal, third-party payer, etc.) and reasons for its selection stated?	4	x	
3.	Were variable estimates used in the analysis from the best available source (i.e., randomized control trial - best, expert opinion - worst)?	8	x	
4.	If estimates came from a subgroup analysis, were the groups pre- specified at the beginning of the study?	1	x (na)	
5.	Was uncertainty handled by (1) statistical analysis to address random events, (2) sensitivity analysis to cover a range of assumptions?	9	x	
6.	Was incremental analysis performed between alternatives for resources and costs?	6	x	
7.	Was the methodology for data abstraction (including the value of health states and other benefits) stated?	5	x	
8.	Did the analytic horizon allow time for all relevant and important outcomes? Were benefits and costs that went beyond 1 year discounted (3% to 5%) and justification given for the discount rate?	7	x	
9.	Was the measurement of costs appropriate and the methodology for the estimation of quantities and unit costs clearly described?	8	x	
10.	Were the primary outcome measure(s) for the economic evaluation clearly stated and did they include the major short-term, long-term, and negative outcomes?	6		x
11.	Were the health outcomes measures/scales valid and reliable? If previously tested valid and reliable measures were not available, was justification given for the measures/scales used?	7	x	
12.	Were the economic model (including structure), study methods and analysis, and the components of the numerator and denominator displayed in a clear, transparent manner?	8	x	
13.	Were the choice of economic model, main assumptions, and limitations of the study stated and justified?	7	x	
14.	Did the author(s) explicitly discuss direction and magnitude of potential biases?	6	x	
15.	Were the conclusions/recommendations of the study justified and based on the study results?	8	x	
16.	Was there a statement disclosing the source of funding for the study?	3	x	
	**TOTAL POINTS**	**100**	94	

12 - The Quality of Health Economic Studies (QHES) instrument – *Zhao, 2024*

No	Questions	Points	Yes	No
1.	Was the study objective presented in a clear, specific, and measurable manner?	7	x	
2.	Were the perspective of the analysis (societal, third-party payer, etc.) and reasons for its selection stated?	4	x	
3.	Were variable estimates used in the analysis from the best available source (i.e., randomized control trial - best, expert opinion - worst)?	8	x	
4.	If estimates came from a subgroup analysis, were the groups pre- specified at the beginning of the study?	1	x	
5.	Was uncertainty handled by (1) statistical analysis to address random events, (2) sensitivity analysis to cover a range of assumptions?	9	x	
6.	Was incremental analysis performed between alternatives for resources and costs?	6	x	
7.	Was the methodology for data abstraction (including the value of health states and other benefits) stated?	5	x	
8.	Did the analytic horizon allow time for all relevant and important outcomes? Were benefits and costs that went beyond 1 year discounted (3% to 5%) and justification given for the discount rate?	7	x	
9.	Was the measurement of costs appropriate and the methodology for the estimation of quantities and unit costs clearly described?	8		x
10.	Were the primary outcome measure(s) for the economic evaluation clearly stated and did they include the major short-term, long-term, and negative outcomes?	6		x
11.	Were the health outcomes measures/scales valid and reliable? If previously tested valid and reliable measures were not available, was justification given for the measures/scales used?	7	x	
12.	Were the economic model (including structure), study methods and analysis, and the components of the numerator and denominator displayed in a clear, transparent manner?	8	x	
13.	Were the choice of economic model, main assumptions, and limitations of the study stated and justified?	7	x	
14.	Did the author(s) explicitly discuss direction and magnitude of potential biases?	6	x	
15.	Were the conclusions/recommendations of the study justified and based on the study results?	8	x	
16.	Was there a statement disclosing the source of funding for the study?	3	x	
	**TOTAL POINTS**	**100**	86	

13 - The Quality of Health Economic Studies (QHES) instrument – *Xia, 2024*

No	Questions	Points	Yes	No
1.	Was the study objective presented in a clear, specific, and measurable manner?	7	x	
2.	Were the perspective of the analysis (societal, third-party payer, etc.) and reasons for its selection stated?	4	x	
3.	Were variable estimates used in the analysis from the best available source (i.e., randomized control trial - best, expert opinion - worst)?	8		x
4.	If estimates came from a subgroup analysis, were the groups pre- specified at the beginning of the study?	1	x (na)	
5.	Was uncertainty handled by (1) statistical analysis to address random events, (2) sensitivity analysis to cover a range of assumptions?	9	x	
6.	Was incremental analysis performed between alternatives for resources and costs?	6	x	
7.	Was the methodology for data abstraction (including the value of health states and other benefits) stated?	5	x	
8.	Did the analytic horizon allow time for all relevant and important outcomes? Were benefits and costs that went beyond 1 year discounted (3% to 5%) and justification given for the discount rate?	7	x	
9.	Was the measurement of costs appropriate and the methodology for the estimation of quantities and unit costs clearly described?	8	x	
10.	Were the primary outcome measure(s) for the economic evaluation clearly stated and did they include the major short-term, long-term, and negative outcomes?	6		x
11.	Were the health outcomes measures/scales valid and reliable? If previously tested valid and reliable measures were not available, was justification given for the measures/scales used?	7	x	
12.	Were the economic model (including structure), study methods and analysis, and the components of the numerator and denominator displayed in a clear, transparent manner?	8	x	
13.	Were the choice of economic model, main assumptions, and limitations of the study stated and justified?	7	x	
14.	Did the author(s) explicitly discuss direction and magnitude of potential biases?	6	x	
15.	Were the conclusions/recommendations of the study justified and based on the study results?	8	x	
16.	Was there a statement disclosing the source of funding for the study?	3	x	
	**TOTAL POINTS**	**100**	86	

14 - The Quality of Health Economic Studies (QHES) instrument – *Kiflen, 2022*

No	Questions	Points	Yes	No
1.	Was the study objective presented in a clear, specific, and measurable manner?	7	x	
2.	Were the perspective of the analysis (societal, third-party payer, etc.) and reasons for its selection stated?	4	x	
3.	Were variable estimates used in the analysis from the best available source (i.e., randomized control trial - best, expert opinion - worst)?	8	x	
4.	If estimates came from a subgroup analysis, were the groups pre- specified at the beginning of the study?	1	x (na)	
5.	Was uncertainty handled by (1) statistical analysis to address random events, (2) sensitivity analysis to cover a range of assumptions?	9	x	
6.	Was incremental analysis performed between alternatives for resources and costs?	6	x	
7.	Was the methodology for data abstraction (including the value of health states and other benefits) stated?	5	x	
8.	Did the analytic horizon allow time for all relevant and important outcomes? Were benefits and costs that went beyond 1 year discounted (3% to 5%) and justification given for the discount rate?	7	x	
9.	Was the measurement of costs appropriate and the methodology for the estimation of quantities and unit costs clearly described?	8	x	
10.	Were the primary outcome measure(s) for the economic evaluation clearly stated and did they include the major short-term, long-term, and negative outcomes?	6	x	
11.	Were the health outcomes measures/scales valid and reliable? If previously tested valid and reliable measures were not available, was justification given for the measures/scales used?	7	x	
12.	Were the economic model (including structure), study methods and analysis, and the components of the numerator and denominator displayed in a clear, transparent manner?	8	x	
13.	Were the choice of economic model, main assumptions, and limitations of the study stated and justified?	7	x	
14.	Did the author(s) explicitly discuss direction and magnitude of potential biases?	6	x	
15.	Were the conclusions/recommendations of the study justified and based on the study results?	8	x	
16.	Was there a statement disclosing the source of funding for the study?	3	x	
	**TOTAL POINTS**	**100**	100	

15 - The Quality of Health Economic Studies (QHES) instrument – *Mujwara, 2022*

No	Questions	Points	Yes	No
1.	Was the study objective presented in a clear, specific, and measurable manner?	7	x	
2.	Were the perspective of the analysis (societal, third-party payer, etc.) and reasons for its selection stated?	4	x	
3.	Were variable estimates used in the analysis from the best available source (i.e., randomized control trial - best, expert opinion - worst)?	8	x	
4.	If estimates came from a subgroup analysis, were the groups pre- specified at the beginning of the study?	1	x (na)	
5.	Was uncertainty handled by (1) statistical analysis to address random events, (2) sensitivity analysis to cover a range of assumptions?	9	x	
6.	Was incremental analysis performed between alternatives for resources and costs?	6	x	
7.	Was the methodology for data abstraction (including the value of health states and other benefits) stated?	5	x	
8.	Did the analytic horizon allow time for all relevant and important outcomes? Were benefits and costs that went beyond 1 year discounted (3% to 5%) and justification given for the discount rate?	7	x	
9.	Was the measurement of costs appropriate and the methodology for the estimation of quantities and unit costs clearly described?	8	x	
10.	Were the primary outcome measure(s) for the economic evaluation clearly stated and did they include the major short-term, long-term, and negative outcomes?	6		x
11.	Were the health outcomes measures/scales valid and reliable? If previously tested valid and reliable measures were not available, was justification given for the measures/scales used?	7	x	
12.	Were the economic model (including structure), study methods and analysis, and the components of the numerator and denominator displayed in a clear, transparent manner?	8	x	
13.	Were the choice of economic model, main assumptions, and limitations of the study stated and justified?	7	x	
14.	Did the author(s) explicitly discuss direction and magnitude of potential biases?	6	x	
15.	Were the conclusions/recommendations of the study justified and based on the study results?	8	x	
16.	Was there a statement disclosing the source of funding for the study?	3	x	
	**TOTAL POINTS**	**100**	94	

16 - The Quality of Health Economic Studies (QHES) instrument *– Mujwara, 2023*

No	Questions	Points	Yes	No
1.	Was the study objective presented in a clear, specific, and measurable manner?	7	x	
2.	Were the perspective of the analysis (societal, third-party payer, etc.) and reasons for its selection stated?	4	x	
3.	Were variable estimates used in the analysis from the best available source (i.e., randomized control trial - best, expert opinion - worst)?	8	x	
4.	If estimates came from a subgroup analysis, were the groups pre- specified at the beginning of the study?	1	x (na)	
5.	Was uncertainty handled by (1) statistical analysis to address random events, (2) sensitivity analysis to cover a range of assumptions?	9	x	
6.	Was incremental analysis performed between alternatives for resources and costs?	6	x	
7.	Was the methodology for data abstraction (including the value of health states and other benefits) stated?	5	x	
8.	Did the analytic horizon allow time for all relevant and important outcomes? Were benefits and costs that went beyond 1 year discounted (3% to 5%) and justification given for the discount rate?	7	x	
9.	Was the measurement of costs appropriate and the methodology for the estimation of quantities and unit costs clearly described?	8	x	
10.	Were the primary outcome measure(s) for the economic evaluation clearly stated and did they include the major short-term, long-term, and negative outcomes?	6		x
11.	Were the health outcomes measures/scales valid and reliable? If previously tested valid and reliable measures were not available, was justification given for the measures/scales used?	7	x	
12.	Were the economic model (including structure), study methods and analysis, and the components of the numerator and denominator displayed in a clear, transparent manner?	8	x	
13.	Were the choice of economic model, main assumptions, and limitations of the study stated and justified?	7	x	
14.	Did the author(s) explicitly discuss direction and magnitude of potential biases?	6	x	
15.	Were the conclusions/recommendations of the study justified and based on the study results?	8	x	
16.	Was there a statement disclosing the source of funding for the study?	3		x
	**TOTAL POINTS**	**100**	91	

17 - The Quality of Health Economic Studies (QHES) instrument – *Martikainen, 2022*

No	Questions	Points	Yes	No
1.	Was the study objective presented in a clear, specific, and measurable manner?	7	x	
2.	Were the perspective of the analysis (societal, third-party payer, etc.) and reasons for its selection stated?	4	x	
3.	Were variable estimates used in the analysis from the best available source (i.e., randomized control trial - best, expert opinion - worst)?	8	x	
4.	If estimates came from a subgroup analysis, were the groups pre- specified at the beginning of the study?	1	x (na)	
5.	Was uncertainty handled by (1) statistical analysis to address random events, (2) sensitivity analysis to cover a range of assumptions?	9	x	
6.	Was incremental analysis performed between alternatives for resources and costs?	6	x	
7.	Was the methodology for data abstraction (including the value of health states and other benefits) stated?	5	x	
8.	Did the analytic horizon allow time for all relevant and important outcomes? Were benefits and costs that went beyond 1 year discounted (3% to 5%) and justification given for the discount rate?	7	x	
9.	Was the measurement of costs appropriate and the methodology for the estimation of quantities and unit costs clearly described?	8	x	
10.	Were the primary outcome measure(s) for the economic evaluation clearly stated and did they include the major short-term, long-term, and negative outcomes?	6		x
11.	Were the health outcomes measures/scales valid and reliable? If previously tested valid and reliable measures were not available, was justification given for the measures/scales used?	7	x	
12.	Were the economic model (including structure), study methods and analysis, and the components of the numerator and denominator displayed in a clear, transparent manner?	8	x	
13.	Were the choice of economic model, main assumptions, and limitations of the study stated and justified?	7	x	
14.	Did the author(s) explicitly discuss direction and magnitude of potential biases?	6	x	
15.	Were the conclusions/recommendations of the study justified and based on the study results?	8	x	
16.	Was there a statement disclosing the source of funding for the study?	3	x	
	**TOTAL POINTS**	**100**	94	

18 - The Quality of Health Economic Studies (QHES) instrument – *Guinan, 2021*

No	Questions	Points	Yes	No
1.	Was the study objective presented in a clear, specific, and measurable manner?	7	x	
2.	Were the perspective of the analysis (societal, third-party payer, etc.) and reasons for its selection stated?	4	x	
3.	Were variable estimates used in the analysis from the best available source (i.e., randomized control trial - best, expert opinion - worst)?	8	x	
4.	If estimates came from a subgroup analysis, were the groups pre- specified at the beginning of the study?	1	x (na)	
5.	Was uncertainty handled by (1) statistical analysis to address random events, (2) sensitivity analysis to cover a range of assumptions?	9	x	
6.	Was incremental analysis performed between alternatives for resources and costs?	6	x	
7.	Was the methodology for data abstraction (including the value of health states and other benefits) stated?	5	x	
8.	Did the analytic horizon allow time for all relevant and important outcomes? Were benefits and costs that went beyond 1 year discounted (3% to 5%) and justification given for the discount rate?	7		x
9.	Was the measurement of costs appropriate and the methodology for the estimation of quantities and unit costs clearly described?	8	x	
10.	Were the primary outcome measure(s) for the economic evaluation clearly stated and did they include the major short-term, long-term, and negative outcomes?	6		x
11.	Were the health outcomes measures/scales valid and reliable? If previously tested valid and reliable measures were not available, was justification given for the measures/scales used?	7	x	
12.	Were the economic model (including structure), study methods and analysis, and the components of the numerator and denominator displayed in a clear, transparent manner?	8	x	
13.	Were the choice of economic model, main assumptions, and limitations of the study stated and justified?	7	x	
14.	Did the author(s) explicitly discuss direction and magnitude of potential biases?	6	x	
15.	Were the conclusions/recommendations of the study justified and based on the study results?	8	x	
16.	Was there a statement disclosing the source of funding for the study?	3	x	
	**TOTAL POINTS**	**100**	87	

19 - The Quality of Health Economic Studies (QHES) instrument – *Liu, 2022*

No	Questions	Points	Yes	No
1.	Was the study objective presented in a clear, specific, and measurable manner?	7	x	
2.	Were the perspective of the analysis (societal, third-party payer, etc.) and reasons for its selection stated?	4	x	
3.	Were variable estimates used in the analysis from the best available source (i.e., randomized control trial - best, expert opinion - worst)?	8	x	
4.	If estimates came from a subgroup analysis, were the groups pre- specified at the beginning of the study?	1	x	
5.	Was uncertainty handled by (1) statistical analysis to address random events, (2) sensitivity analysis to cover a range of assumptions?	9	x	
6.	Was incremental analysis performed between alternatives for resources and costs?	6	x	
7.	Was the methodology for data abstraction (including the value of health states and other benefits) stated?	5	x	
8.	Did the analytic horizon allow time for all relevant and important outcomes? Were benefits and costs that went beyond 1 year discounted (3% to 5%) and justification given for the discount rate?	7	x	
9.	Was the measurement of costs appropriate and the methodology for the estimation of quantities and unit costs clearly described?	8	x	
10.	Were the primary outcome measure(s) for the economic evaluation clearly stated and did they include the major short-term, long-term, and negative outcomes?	6		x
11.	Were the health outcomes measures/scales valid and reliable? If previously tested valid and reliable measures were not available, was justification given for the measures/scales used?	7	x	
12.	Were the economic model (including structure), study methods and analysis, and the components of the numerator and denominator displayed in a clear, transparent manner?	8	x	
13.	Were the choice of economic model, main assumptions, and limitations of the study stated and justified?	7	x	
14.	Did the author(s) explicitly discuss direction and magnitude of potential biases?	6	x	
15.	Were the conclusions/recommendations of the study justified and based on the study results?	8	x	
16.	Was there a statement disclosing the source of funding for the study?	3	x	
	**TOTAL POINTS**	**100**	94	

20 - The Quality of Health Economic Studies (QHES) instrument – *Berdunov, 2024*

No	Questions	Points	Yes	No
1.	Was the study objective presented in a clear, specific, and measurable manner?	7	x	
2.	Were the perspective of the analysis (societal, third-party payer, etc.) and reasons for its selection stated?	4	x	
3.	Were variable estimates used in the analysis from the best available source (i.e., randomized control trial - best, expert opinion - worst)?	8	x	
4.	If estimates came from a subgroup analysis, were the groups pre- specified at the beginning of the study?	1	x (na)	
5.	Was uncertainty handled by (1) statistical analysis to address random events, (2) sensitivity analysis to cover a range of assumptions?	9	x	
6.	Was incremental analysis performed between alternatives for resources and costs?	6	x	
7.	Was the methodology for data abstraction (including the value of health states and other benefits) stated?	5	x	
8.	Did the analytic horizon allow time for all relevant and important outcomes? Were benefits and costs that went beyond 1 year discounted (3% to 5%) and justification given for the discount rate?	7	x	
9.	Was the measurement of costs appropriate and the methodology for the estimation of quantities and unit costs clearly described?	8	x	
10.	Were the primary outcome measure(s) for the economic evaluation clearly stated and did they include the major short-term, long-term, and negative outcomes?	6		x
11.	Were the health outcomes measures/scales valid and reliable? If previously tested valid and reliable measures were not available, was justification given for the measures/scales used?	7	x	
12.	Were the economic model (including structure), study methods and analysis, and the components of the numerator and denominator displayed in a clear, transparent manner?	8	x	
13.	Were the choice of economic model, main assumptions, and limitations of the study stated and justified?	7	x	
14.	Did the author(s) explicitly discuss direction and magnitude of potential biases?	6	x	
15.	Were the conclusions/recommendations of the study justified and based on the study results?	8	x	
16.	Was there a statement disclosing the source of funding for the study?	3	x	
	**TOTAL POINTS**	**100**	94	

21 - The Quality of Health Economic Studies (QHES) instrument – *Kelemen, 2024*

No	Questions	Points	Yes	No
1.	Was the study objective presented in a clear, specific, and measurable manner?	7	x	
2.	Were the perspective of the analysis (societal, third-party payer, etc.) and reasons for its selection stated?	4	x	
3.	Were variable estimates used in the analysis from the best available source (i.e., randomized control trial - best, expert opinion - worst)?	8	x	
4.	If estimates came from a subgroup analysis, were the groups pre- specified at the beginning of the study?	1	x (na)	
5.	Was uncertainty handled by (1) statistical analysis to address random events, (2) sensitivity analysis to cover a range of assumptions?	9	x	
6.	Was incremental analysis performed between alternatives for resources and costs?	6	x	
7.	Was the methodology for data abstraction (including the value of health states and other benefits) stated?	5	x	
8.	Did the analytic horizon allow time for all relevant and important outcomes? Were benefits and costs that went beyond 1 year discounted (3% to 5%) and justification given for the discount rate?	7		x
9.	Was the measurement of costs appropriate and the methodology for the estimation of quantities and unit costs clearly described?	8	x	
10.	Were the primary outcome measure(s) for the economic evaluation clearly stated and did they include the major short-term, long-term, and negative outcomes?	6		x
11.	Were the health outcomes measures/scales valid and reliable? If previously tested valid and reliable measures were not available, was justification given for the measures/scales used?	7	x	
12.	Were the economic model (including structure), study methods and analysis, and the components of the numerator and denominator displayed in a clear, transparent manner?	8	x	
13.	Were the choice of economic model, main assumptions, and limitations of the study stated and justified?	7	x	
14.	Did the author(s) explicitly discuss direction and magnitude of potential biases?	6	x	
15.	Were the conclusions/recommendations of the study justified and based on the study results?	8	x	
16.	Was there a statement disclosing the source of funding for the study?	3	x	
	**TOTAL POINTS**	**100**	87	

22 - The Quality of Health Economic Studies (QHES) instrument – *Vernon, 2024*

No	Questions	Points	Yes	No
1.	Was the study objective presented in a clear, specific, and measurable manner?	7	x	
2.	Were the perspective of the analysis (societal, third-party payer, etc.) and reasons for its selection stated?	4		x
3.	Were variable estimates used in the analysis from the best available source (i.e., randomized control trial - best, expert opinion - worst)?	8	x	
4.	If estimates came from a subgroup analysis, were the groups pre- specified at the beginning of the study?	1	x (na)	
5.	Was uncertainty handled by (1) statistical analysis to address random events, (2) sensitivity analysis to cover a range of assumptions?	9		x
6.	Was incremental analysis performed between alternatives for resources and costs?	6		x
7.	Was the methodology for data abstraction (including the value of health states and other benefits) stated?	5	x	
8.	Did the analytic horizon allow time for all relevant and important outcomes? Were benefits and costs that went beyond 1 year discounted (3% to 5%) and justification given for the discount rate?	7		x
9.	Was the measurement of costs appropriate and the methodology for the estimation of quantities and unit costs clearly described?	8	x	
10.	Were the primary outcome measure(s) for the economic evaluation clearly stated and did they include the major short-term, long-term, and negative outcomes?	6		x
11.	Were the health outcomes measures/scales valid and reliable? If previously tested valid and reliable measures were not available, was justification given for the measures/scales used?	7	x	
12.	Were the economic model (including structure), study methods and analysis, and the components of the numerator and denominator displayed in a clear, transparent manner?	8	x	
13.	Were the choice of economic model, main assumptions, and limitations of the study stated and justified?	7	x	
14.	Did the author(s) explicitly discuss direction and magnitude of potential biases?	6		x
15.	Were the conclusions/recommendations of the study justified and based on the study results?	8	x	
16.	Was there a statement disclosing the source of funding for the study?	3	x	
	**TOTAL POINTS**	**100**	62	

23 - The Quality of Health Economic Studies (QHES) instrument – *Yang, 2024*

No	Questions	Points	Yes	No
1.	Was the study objective presented in a clear, specific, and measurable manner?	7	x	
2.	Were the perspective of the analysis (societal, third-party payer, etc.) and reasons for its selection stated?	4	x	
3.	Were variable estimates used in the analysis from the best available source (i.e., randomized control trial - best, expert opinion - worst)?	8	x	
4.	If estimates came from a subgroup analysis, were the groups pre- specified at the beginning of the study?	1	x (na)	
5.	Was uncertainty handled by (1) statistical analysis to address random events, (2) sensitivity analysis to cover a range of assumptions?	9	x	
6.	Was incremental analysis performed between alternatives for resources and costs?	6	x	
7.	Was the methodology for data abstraction (including the value of health states and other benefits) stated?	5	x	
8.	Did the analytic horizon allow time for all relevant and important outcomes? Were benefits and costs that went beyond 1 year discounted (3% to 5%) and justification given for the discount rate?	7	x	
9.	Was the measurement of costs appropriate and the methodology for the estimation of quantities and unit costs clearly described?	8	x	
10.	Were the primary outcome measure(s) for the economic evaluation clearly stated and did they include the major short-term, long-term, and negative outcomes?	6		x
11.	Were the health outcomes measures/scales valid and reliable? If previously tested valid and reliable measures were not available, was justification given for the measures/scales used?	7	x	
12.	Were the economic model (including structure), study methods and analysis, and the components of the numerator and denominator displayed in a clear, transparent manner?	8	x	
13.	Were the choice of economic model, main assumptions, and limitations of the study stated and justified?	7	x	
14.	Did the author(s) explicitly discuss direction and magnitude of potential biases?	6	x	
15.	Were the conclusions/recommendations of the study justified and based on the study results?	8	x	
16.	Was there a statement disclosing the source of funding for the study?	3	x	
	**TOTAL POINTS**	**100**	94	

24 - The Quality of Health Economic Studies (QHES) instrument – *Jiang, 2024*

No	Questions	Points	Yes	No
1.	Was the study objective presented in a clear, specific, and measurable manner?	7	x	
2.	Were the perspective of the analysis (societal, third-party payer, etc.) and reasons for its selection stated?	4	x	
3.	Were variable estimates used in the analysis from the best available source (i.e., randomized control trial - best, expert opinion - worst)?	8	x	
4.	If estimates came from a subgroup analysis, were the groups pre- specified at the beginning of the study?	1	x (na)	
5.	Was uncertainty handled by (1) statistical analysis to address random events, (2) sensitivity analysis to cover a range of assumptions?	9	x	
6.	Was incremental analysis performed between alternatives for resources and costs?	6	x	
7.	Was the methodology for data abstraction (including the value of health states and other benefits) stated?	5	x	
8.	Did the analytic horizon allow time for all relevant and important outcomes? Were benefits and costs that went beyond 1 year discounted (3% to 5%) and justification given for the discount rate?	7	x	
9.	Was the measurement of costs appropriate and the methodology for the estimation of quantities and unit costs clearly described?	8	x	
10.	Were the primary outcome measure(s) for the economic evaluation clearly stated and did they include the major short-term, long-term, and negative outcomes?	6		x
11.	Were the health outcomes measures/scales valid and reliable? If previously tested valid and reliable measures were not available, was justification given for the measures/scales used?	7	x	
12.	Were the economic model (including structure), study methods and analysis, and the components of the numerator and denominator displayed in a clear, transparent manner?	8	x	
13.	Were the choice of economic model, main assumptions, and limitations of the study stated and justified?	7	x	
14.	Did the author(s) explicitly discuss direction and magnitude of potential biases?	6	x	
15.	Were the conclusions/recommendations of the study justified and based on the study results?	8	x	
16.	Was there a statement disclosing the source of funding for the study?	3	x	
	**TOTAL POINTS**	**100**	94	
